# Reduction in Olfactory Discomfort in Inhabited Premises from Areas with Mofettas through Cellulosic Derivative–Polypropylene Hollow Fiber Composite Membranes

**DOI:** 10.3390/ma17174437

**Published:** 2024-09-09

**Authors:** Paul Constantin Albu, Andreia Pîrțac, Ludmila Motelica, Aurelia Cristina Nechifor, Geani Teodor Man, Alexandra Raluca Grosu, Szidonia-Katalin Tanczos, Vlad-Alexandru Grosu, Gheorghe Nechifor

**Affiliations:** 1 Radioisotopes and Radiation Metrology Department (DRMR), National Institute of Physics and Nuclear Engineering (IFIN) Horia Hulubei, 023465 Măgurele, Romania; paulalbu@gmail.com; 2 Analytical Chemistry and Environmental Engineering Department, National University of Science and Technology POLITEHNICA Bucharest, 011061 Bucharest, Romania; andreia.pascu@yahoo.ro (A.P.); aureliacristinanechifor@gmail.com (A.C.N.); geani.man@icsi.ro (G.T.M.); andra.grosu@upb.ro (A.R.G.); 3 National Research Center for Micro and Nanomaterials, Department of Science and Engineering of Nanomaterials and Oxide Materials, National University of Science and Technology POLITEHNICA Bucharest, 060042 Bucharest, Romania; ludmila.motelica@upb.ro; 4 National Research and Development Institute for Cryogenics and Isotopic Technologies—ICSI, 240050 Râmnicu-Vâlcea, Romania; 5 Department of Bioengineering, University Sapientia of Miercurea-Ciuc, 500104 Miercurea-Ciuc, Romania; tczszidonia@yahoo.com; 6 Department of Electronic Technology and Reliability, Faculty of Electronics, Telecommunications and Information Technology, National University of Science and Technology POLITEHNICA Bucharest, 061071 Bucharest, Romania

**Keywords:** hydrogen sulfide separation, polluted air, composite membranes, sodium carboxymethyl–cellulose, cellulose acetate, methyl 2–hydroxyethyl–cellulose, hydroxyethyl–cellulose

## Abstract

Hydrogen sulfide is present in active or extinct volcanic areas (mofettas). The habitable premises in these areas are affected by the presence of hydrogen sulfide, which, even in low concentrations, gives off a bad to unbearable smell. If the living spaces considered are closed enclosures, then a system can be designed to reduce the concentration of hydrogen sulfide. This paper presents a membrane-based way to reduce the hydrogen sulfide concentration to acceptable limits using a cellulosic derivative–propylene hollow fiber-based composite membrane module. The cellulosic derivatives considered were: carboxymethyl–cellulose (NaCMC), P1; cellulose acetate (CA), P2; methyl 2–hydroxyethyl–cellulose (MHEC), P3; and hydroxyethyl–cellulose (HEC), P4. In the permeation module, hydrogen sulfide is captured with a solution of cadmium that forms cadmium sulfide, usable as a luminescent substance. The composite membranes were characterized by SEM, EDAX, FTIR, FTIR 2D maps, thermal analysis (TG and DSC), and from the perspective of hydrogen sulfide air removal performance. To determine the process performances, the variables were as follows: the nature of the cellulosic derivative–polypropylene hollow fiber composite membrane, the concentration of hydrogen sulfide in the polluted air, the flow rate of polluted air, and the pH of the cadmium nitrate solution. The pertraction efficiency was highest for the sodium carboxymethyl–cellulose (NaCMC)–polypropylene hollow fiber membrane, with a hydrogen sulfide concentration in the polluted air of 20 ppm, a polluted air flow rate (Q_H2S_) of 50 L/min, and a pH of 2 and 4. The hydrogen sulfide flux rates, for membrane P1, fall between 0.25 × 10^−7^ mol·m^2^·s^−1^ for the values of Q_H2S_ = 150 L/min, C_H2S_ = 20 ppm, and pH = 2 and 0.67 × 10^−7^ mol·m^−2^·s^−1^ for the values of Q_H2S_ = 50 L/min, C_H2S_ = 60 ppm, and pH = 2. The paper proposes a simple air purification system containing hydrogen sulfide, using a module with composite cellulosic derivative–polypropylene hollow fiber membranes.

## 1. Introduction

Among the nonmetal hydrides (NMH_n_) only water is liquid and, of course, non-toxic. The other hydrides of nonmetals are gasses with variable toxicity [1,2,3]. Of these, hydrogen sulfide is particularly important, which can appear in the environment both naturally (active or extinct volcanoes) [4,5,6,7] and as a result of human activity [8,9,10,11,12,13,14,15]. Among the sources originating from human activities, most notable are anaerobic decomposition [8], nitrogenous fertilizers [9], treatment plants [10], effluents of treatment plants [11], dye and dye intermediates, [12], sugar and distilleries [13], pesticides [14], and pulp and paper limekiln [15].

Hydrogen sulfide is undesirable both in industrial processes (corrosion being the main reason) [16,17] and in contact with humans (toxicity being the predominant reason) [18,19,20].

**Figure 1:**
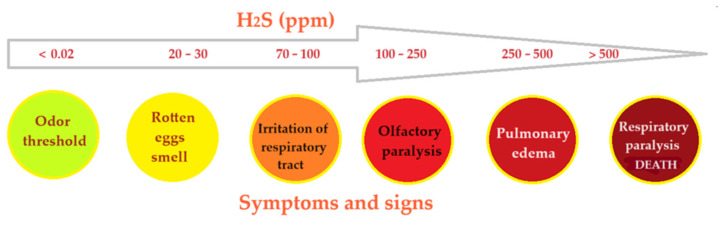
The effects of hydrogen sulfide on human beings as a function of its concentration in the air.

One of the ways of interpreting the effect of hydrogen sulfide in contact with humans is shown in Figure 1 [21,22].

Considering the undesirable effects both on industrial installations and on humans, researchers have focused on the elimination of hydrogen sulfide or its derivatives (mercaptans, thiophene, etc.) from various gaseous or liquid environments [23,24,25].

There are various ways of removing hydrogen sulfide: absorption [26], adsorption [27], electrochemical degradation [28], photochemical degradation [29], catalytic degradation [30], or biodegradation [31,32,33]. All the presented elimination processes are non-regenerative, and the resulting products are sulfur, in reductive processes, and sulfur oxides in oxidative processes [34,35].

One of the ways to remove hydrogen sulfide is represented by membrane processes, which can be reducing or oxidizing, but also recuperative, with hydrogen sulfide being fixed in compounds with practical utility [36,37,38,39,40,41,42]. Membrane techniques use polymeric [43], inorganic [44], composite [45], or liquid [46,47,48] membranes.

The studies in the specialized literature were carried out with bulk liquid membranes (BLM), emulsion liquid membranes (ELM), and hollow fiber membranes (HFM) [36,37,38,39,40,41,42,43,44,45,46,47,48].

However, for the present study regarding the reduction in hydrogen sulfide concentration in closed habitable premises, two types of membranes cannot be taken into account. These are BLMs—which require a high volume of membrane solvent being dedicated to laboratory studies, and ELMs—which are dedicated to industrial applications because they have a large, but uncontrollable, contact surface [38,39,44,49,50].

The hollow fiber membranes are the only ones that can be considered for this study because they have a large and stable contact surface (compared to ELMs) and require a low volume of selective compound or membrane solvent [37,42,44,46,47,48,50,51,52].

The applications of membranes and membrane techniques are in continuous development, being applied both in the separation processes of dispersed systems and gas mixtures [49,50,51,52,53,54]. In the last decade, biopolymers such as chitosan or cellulosic derivatives present in various composite membranes have been tested in sulfur gas separation processes [55,56,57,58].

In this paper, we start from the results obtained previously [57,58,59] and study composite polypropylene hollow fiber–cellulosic derivatives (sodium carboxymethyl–cellulose, cellulose acetate, 2–methyl–hydroxyethyl–cellulose, and hydroxy–ethyl–cellulose); hydrogen sulfide is recuperatively separated from the gas mixture, being fixed as cadmium sulfide in the composite membrane module.

This paper approaches the separation of hydrogen sulfide in low concentrations (20–60 ppm) from closed habitable premises in isolated areas where hydrogen sulfide is naturally produced (mofettas). The system for reducing the content of hydrogen sulfide to acceptable limits is based on HF (hollow-fiber) composite membranes with a polypropylene matrix and selective compounds derived of cellulose. The new system involves both the capture of hydrogen sulfide as cadmium sulfide, as well as the exploitation of the membrane module by melting and obtaining reflective material.

## 2. Materials and Methods

### 2.1. Materials

The materials used in the present work were of analytical purity. They were purchased from Merck (Merck KGaA, Darmstadt, Germany): Cd (NO_3_)_2_ tetrahydrate (308.48 g/mol), dimethyl formamide (DMF), sodium sulfide (Na_2_S) [78.0452 g/mol (anhydrous)], hydrogen sulfide, sodium hydroxide, and nitric acid [58].

The cellulosic derivatives—sodium carboxymethyl–cellulose (NaCMC), cellulose acetate (CA), methyl 2–hydroxyethyl–cellulose (MHEC), and hydroxyethyl–cellulose (HEC) (Table 1) [59]—were purchased from Sigma-Aldrich (Merck KGaA, Darmstadt, Germany).

The tubular dialysis membranes were provided by Visking (Medicell Membranes Ltd., London, UK) [57].

An MQuant^®^ sulfide test (Merck Millipore from Merck KGaA, Darmstadt, Germany), and a Sulfide Test photometric Spectroquant^®^ (Merck KGaA, Darmstadt, Germany) were used [58].

The hollow fiber polypropylene support membranes (PPSM) were provided by GOST Ltd., Perugia, Italy [60].

The purified water, characterized by a conductivity of 18.2 µS/cm, was obtained with an RO Millipore system (MilliQ^®^ Direct 8 RO Water Purification System, Merck, Darmstadt, Germany) [61,62].

### 2.2. Preparation of Cellulosic Derivatives–Polypropylene Hollow Fiber Membrane

The cellulosic derivatives (NaCMC, CA, MHEC, and HEC) with the characteristics indicated in Table 1 were solubilized in a mass concentration of 4%, in dimethylformamide without traces of water. For this, the glass vessel, in which the dimethylformamide and the corresponding amount of polymer were introduced, is closed tightly (the possibility of absorbing water vapor from the working atmosphere is eliminated) and placed in an ultrasonic bath (Elmasonic S, Elma Schmidbauer GmbH, Singen, Germany) for 24 h, thus observing the complete solubilization and obtaining the polymeric dispersion [63]. The obtained solutions are subjected to centrifugation, then they are kept in closed glass vessels for 24 h to remove bubbles.

**Figure 2:**
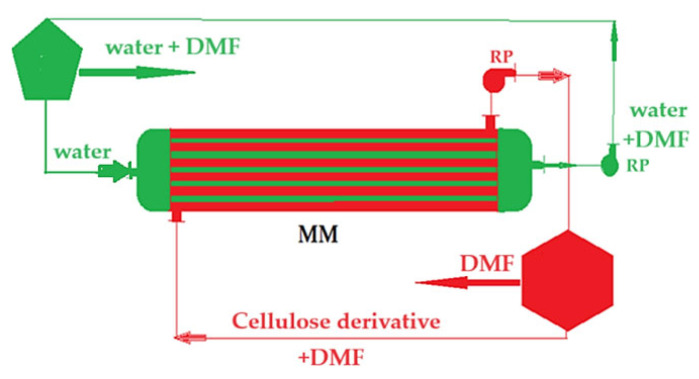
A schematic presentation of the installation for the preparation of the composite membrane: MM—membrane module; DMF—dimethyl formamide; RP—recirculation pumps [64].

Obtaining cellulosic derivative–polypropylene hollow fiber composite membranes is carried out in the module shown in Figure 2 [64] as follows:

- The solution of cellulosic derivative in dimethylformamide (DMF) is introduced through the outside of the polypropylene hollow fibers in the membrane module (MM); the volume of the polymer solution used is 2 L, recirculated at a flow rate of 100 mL/min for a hollow-fiber module surface of 0.1 m^2^;
- Water is introduced through the inside of the polypropylene hollow fibers. A volume of 5 L of pure water is recirculated at a flow rate of 250 mL/min;
- The two phases are contacted in the membrane module, resulting in the composite membrane by phase inversion [61], cellulosic derivative dispersion in DMF, and aqueous DMF solution;
- After carrying out the obtaining procedure, the membranes are washed with pure water, 10 L of pure water, with a flow rate of 500 mL/min, by introducing water between the cellulosic derivative–polypropylene hollow fiber composite membranes;
- Four types of composite membranes were obtained, shown in Table 1 (P1, P2, P3, and P4).

After retaining the hydrogen sulfide in the cadmium solution following the permeation of the polluted air with hydrogen sulfide, for 24 h, on the P1 membrane, the cadmium sulfide membrane (P5) is obtained. The P5 membrane has a sulfide layer deposited on the sodium carboxymethyl–cellulose (NaCMC) hollow fiber membrane (P1) surface, and is characterized by SEM and EDAX.

Wet cadmium sulfide is recovered from the composite membranes, resulting in sample P6.

### 2.3. Permeation Procedures

**Figure 3:**
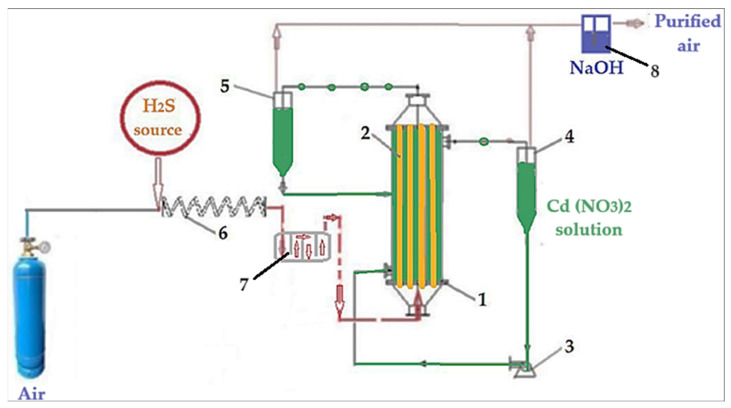
A schematic presentation of the laboratory installation for hydrogen sulfide sequestration from a gaseous mixture: 1—membrane contactor; 2—composite hollow fiber membranes; 3—pump for metal ion acidic solutions; 4 and 5—gas–liquid separator; 6—homogenization; 7—slow flow module; 8—sodium hydroxide hatch [58].

The composite membranes thus obtained were characterized by scanning electron microscopy (SEM), Energy Dispersive X–ray Spectroscopy (EDAX), thermal analysis (TG and DSC), Fourier Transform Infra-Red spectroscopy (FTIR), and FTIR 2D maps, in preparation for the permeation tests of the air–hydrogen sulfide gas system (Figure 3) [58].

The separation installation of hydrogen sulfide from air (Figure 3) works like this:

- The gaseous mixture is made by dispersing hydrogen sulfide coming from a source that allows a precise dose of ±0.1% in volumetric percentages, by mixing with air dosed in the specific bottle;
- After mixing, the air containing hydrogen sulfide is homogenized by passing through a 5.0 m serpentine (6) and slows down in chamber 7;
- The air polluted with hydrogen sulfide is introduced into the permeation module (1) through the composite membranes (2);
- The cadmium nitrate solution (receiving phase) is introduced through the outside of the composite membranes using pump 3;
- Separators 4 and 5 collect any gaseous mixture that will be captured in hatch 8 with sodium hydroxide.

The pertraction efficiency (*PE*%) for the species of interest (hydrogen sulfide) using the concentration of the solutions [65] was calculated as follows, as in Equation (1):

(1) $$PE\;(\%)=(c_{0}-c_{f})/c_{0}\times 100$$

with *c_f_* being the final concentration of the solute (hydrogen sulfide) and *c*_0_ the initial concentration of solute (hydrogen sulfide).

The flux of permeated hydrogen sulfide (*J*) is calculated based on Relation (2):

(2) $$J=\;m/(A\times \Delta t)\cdot (\mathrm{mol}\times m^{-2}\times s^{-1})$$

where *m* is the mass of hydrogen sulfide (mol), *A* is the effective membrane area (m^2^), and Δt is the processing time interval (s).

The measurements were made using MQuant^®^ sulfide test (Merck Millipore from Merck KGaA, Darmstadt, Germany), and the photometric sulfide test Spectroquant^®^ (Merck KGaA, Darmstadt, Germany) [57].

The measurements were independently validated using an Oldham MX 21 gas detector (MX 21 Plus Multigas, Arras, France) equipped with electrochemical sensors, and a H_2_S Model 3000RS Analyzer (MultiLab LLC, Bucharest, Romania) [58].

Analytic measurements, with a number of five for each sample taken, were performed for three independent hydrogen sulfide retention experiments. The evaluation of the analytical data was performed by taking the arithmetic mean of those five analytical values obtained for each point. To choose the most suitable membrane for the study, the errors were determined as percentages.

The standard error was used for the pertraction efficiency (PE) graphs as a function of the hydrogen sulfide flow rate or concentration. The data processing mode took into account the propagation of errors due to the control of the operating parameters (flow rate, pH, concentration) as well as those determined by sampling.

### 2.4. Equipment

Microscopy tests, scanning electron microscopy (SEM), and high-resolution SEM (HR SEM), were performed using a Hitachi S4500 system (Hitachi High-Technologies Europe GmbH, Mannheim, Germany). Composite membrane samples with a length of 3 cm were fractured in liquid nitrogen and metallized with a superficial layer of gold, to allow the surface and the section of the membranes to be analyzed by scanning electron microscopy (SEM) coupled with energy-dispersive X-ray analysis (EDAX), with a Hitachi S4500 system [58,66].

Thermal characterizations were carried out using a Netzsch STA 449C Jupiter apparatus (NETZSCH-Gerätebau GmbH, Selb, Germany). Each sample was weighed as approximatively 10 mg. The samples were placed in an open alumina crucible and heated up to 900 °C with a 10 K∙min^−1^ rate, under a flow of 50 mL∙min^−1^ dried air. As a reference, we used an empty alumina crucible. The evolved gasses were analyzed with an FTIR Tensor 27 from Bruker (Bruker Co., Ettlingen, Germany), equipped with a thermostat gas cell [57,58,62].

A Spectroscopy Bruker Tensor 27 FTIR with a Diamond Attenuated Total Reflection—ATR (Bruker)—was used for spectrometric study in the range of 500 to 4000 cm^−1^ [59].

FTIR 2D maps were recorded with a Nicolet iS50R FTIR microscope and Nicolet iZ10 Module Part (Thermo Fisher Scientific Inc., Waltham, MA, USA), with a DTGS detector, in the wavenumber range 4000–600 cm^−1^ [67].

UV-VIS analysis was performed on a Spectrometer CamSpec M550 (Spectronic CamSpec Ltd., Leeds, UK) [66].

The pH of the medium was tested with a combined selective electrode (HI 4107, Hanna Instruments Ltd., Leighton Buzzard, UK) and a multi-parameter system (HI 5522, Hanna Instruments Ltd., Leighton Buzzard, UK) [57,58].

Another device used was an ultrasonic bath (Elmasonic S, Elma Schmidbauer GmbH, Singen, Germany) [61].

## 3. Results and Discussion

Closed habitable spaces in areas with natural hydrogen sulfide emanation (mofettas, volcanic areas) were not considered for artificial improvement of breathable air. The only method used was to disperse the polluted air and refresh it with new air. However, the natural background was close to the limit of respiratory tract irritation, and therefore a more effective measure would be to close the respective spaces and treat the air using known methods, of which adsorption or absorption would be the most convenient.

The direct retention of hydrogen sulfide was not taken into account because, along with the hydrogen sulfide, there was also a high percentage of carbon dioxide in the air, which would produce carbonate and hydroxy-carbonates, thus consuming the cadmium from the solution. For this reason, the direct retention of hydrogen sulfide through a contactor was not addressed either. In order to solve the competition between the formation of cadmium sulfide and cadmium carbonates, two parameters were chosen: one regarding the membrane which is composite and selective, and the second related to the pH which was chosen in an acidic environment (favoring the formation of cadmium carbonates).

In the present paper, we propose a new way of retaining hydrogen sulfide from closed habitable premises by using membrane-based permeators and the hydrogen sulfide fixation reaction with cadmium ions in acidic aqueous solution (3):

Cd^2+^ + 2H_2_O + H_2_S → CdS + 2H_3_O^+^ (3)

The solubility product (*K_s_*) in Relation (4) depends on the pH due to the basicity of the sulfide anion (*S*^2−^) [57,58]:

(4) $$\left(K_{s})=[M^{2-}][S^{2-}\right]$$

The acidic aqueous solution, chosen in the present study, does not affect the formation of cadmium sulfide, which is stable in acid medium up to pH 2 [58].

The composite membranes proposed to be used in the permeation module (Figure 3) are obtained by impregnating polypropylene hollow fiber membrane supported with cellulosic derivatives by the method illustrated in Figure 2.

The obtained composite membranes required morphological, structural characterization and the determination of process performances in the retention of hydrogen sulfide in the aqueous solution of cadmium ions.

Morphological characterization was carried out by scanning electron microscopy (SEM) and two-dimensional Fourier Transform InfraRed maps (2D FTIR maps).

The compositional characterization was carried out by analyzing the surface of the composite membranes, before and after the hydrogen sulfide retention process, by Energy Dispersive X-ray Spectroscopy (EDAX) and in the whole membrane by FTIR.

A special part of the study is thermal gravimetric analysis (TG) and differential calorimetric scanning (DSC), as it proposed to use the membranes used in the process of making reflective road markings.

### 3.1. Morphological and Structural Characterization

#### 3.1.1. Morphological Characterization

In order to determine the in-section and on-surface morphology of the composite membranes, one centimeter of the cellulosic derivative–polypropylene hollow fiber composite membrane was prepared, which was fractured and covered with a thin film (approx. 50 nm) of gold.

**Figure 4:**
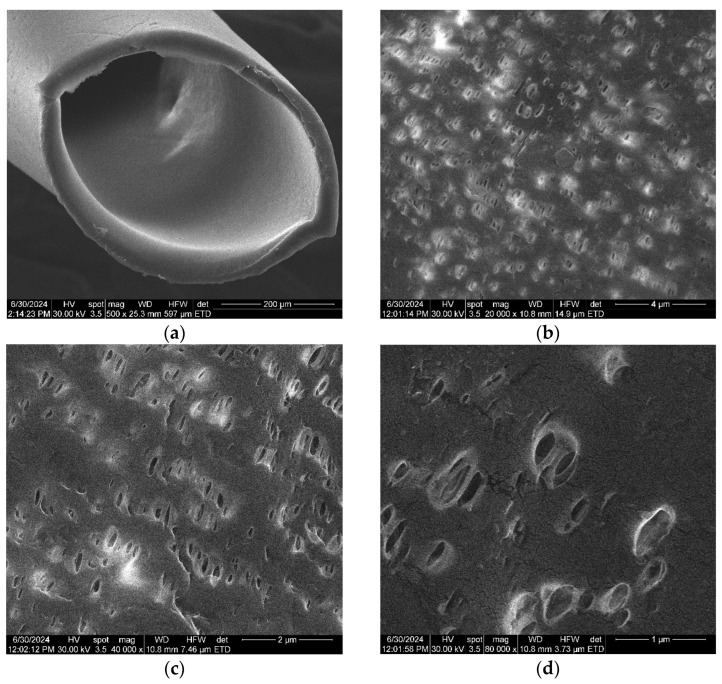

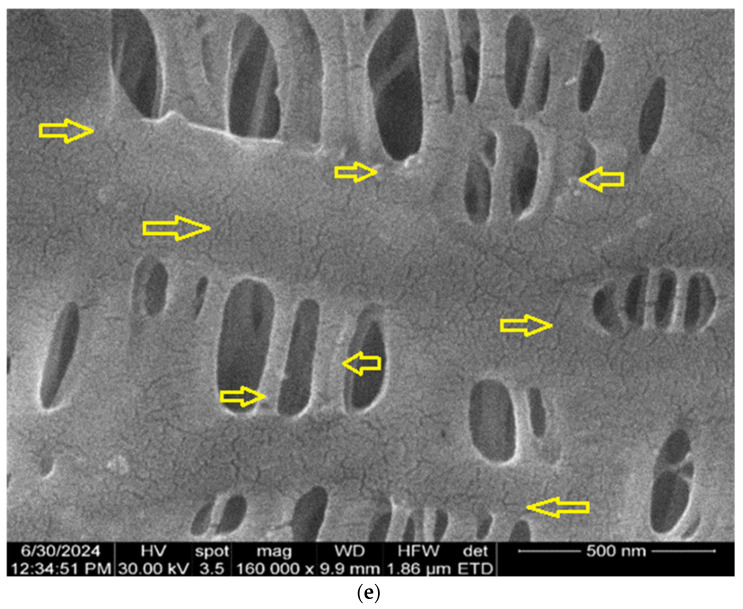
Scanning electron microscopy for sodium carboxymethyl–cellulose–polypropylene hollow fiber membrane (P1): (**a**) composite membrane section; (**b**) membrane surface at 20,000× magnification; (**c**) membrane surface at 40,000× magnification; (**d**) membrane surface at 80,000× magnification; and (**e**) membrane surface at 160,000× magnification.

The diameter of sodium carboxymethyl–cellulose–polypropylene hollow fiber membrane (P1) (Figure 4a) is about 350 µm, with walls of approx. 20–30 µm, confirming the data obtained previously [57,58]. The surface of the composite membrane (Figure 4b–d) shows relatively evenly distributed pores with a diameter of about 1–2 µm. Figure 4d,e show the coverage of the polypropylene hollow fiber support with the cellulosic derivative, both on the surface of the fiber and inside the pores. The obtained data can also be observed for samples P2, P3, and P4 (see Figures S1–S3 in the Supplementary Materials).

Figure 4e shows nano-cracks and nano-clusters (yellow arrows) specific to a wet material, after drying. SEM determinations are made under vacuum, so any wet material is dried. This aspect emphasizes the formation of a hydrophilic film of sodium carboxymethyl–cellulose. This fact is relevant for the formation of the active layer of cellulosic derivative (see also Figures S1, S2, and S4), knowing that polypropylene is hydrophobic.

**Figure 5:**
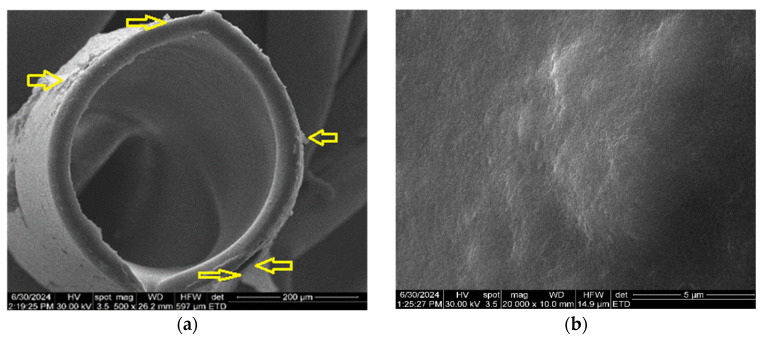

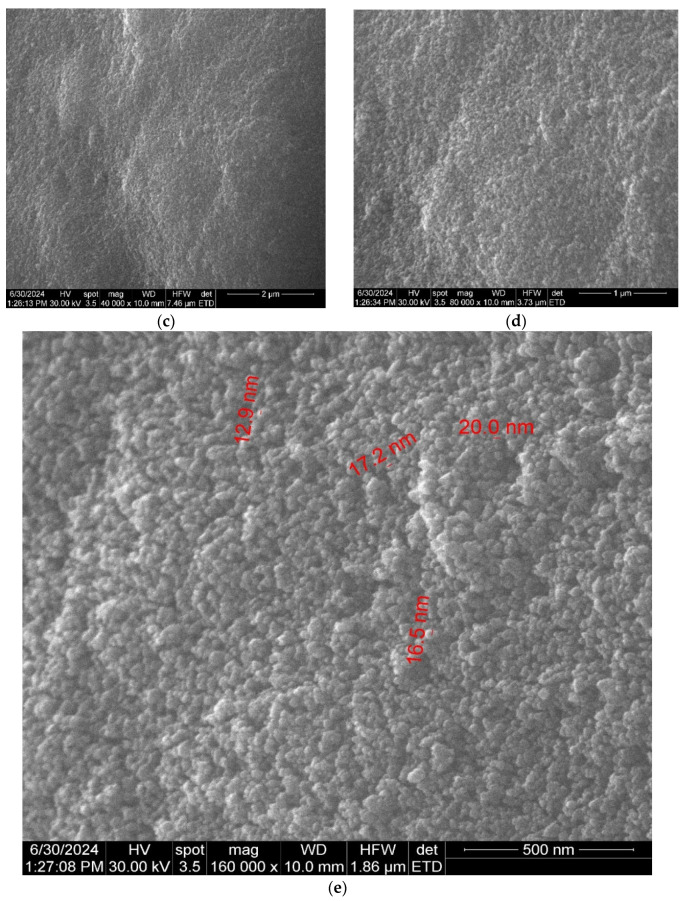
Scanning electron microscopy for sodium carboxymethyl–cellulose–polypropylene hollow fiber membrane after using hydrogen sulfide in the separation process and capturing as cadmium sulfide (P5): (**a**) composite membrane section; (**b**) membrane surface at 20,000× magnification; (**c**) membrane surface at 40,000× magnification; (**d**) membrane surface at 80,000× magnification; and (**e**) membrane surface at 160,000× magnification.

After use in the hydrogen sulfide separation process and capture as cadmium sulfide on the sodium carboxymethyl–cellulose–polypropylene hollow fiber membrane (P5), SEM was performed for the membrane section (Figure 5a) in which the layer of cadmium sulfide can be observed (yellow arrows).

On the surface of the ‘sodium carboxymethyl–cellulose–polypropylene hollow fiber’ membrane (P5) the sizes of cadmium sulfide nanoparticles are present (Figure 5b–e). At a 160,000× resolution cadmium sulfide nanoparticles are measurable (Figure 5e).

In Figure 5a, which represents the section of the P5 membrane, the cadmium sulfide layer is highlighted with yellow arrows. This layer was examined at various magnitudes (Figure 5b–e). The nanometric size of the cadmium sulfide particles deposited on the P1 membrane is indicated by the measurable values (numbers written in red).

#### 3.1.2. Structural Characterization

**Figure 6:**
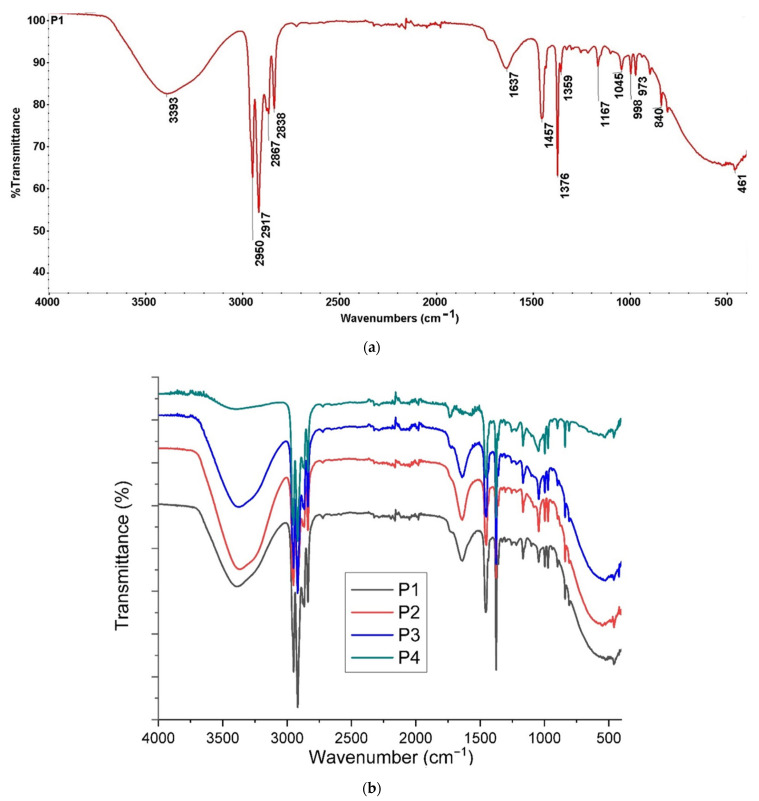
Fourier Transform Infra-Red (FTIR) spectra for (**a**) composite membrane P1 and (**b**) the four samples P1, P2, P3, and P4.

For sodium carboxymethyl–cellulose–polypropylene hollow fiber membrane, the FTIR spectrum was obtained (Figure 6a), which allowed the choice of wave numbers for the creation of 2D maps. As can be seen in Figure 6b, the spectra of the four composite membranes (P1, P2, P3, and P4) are relatively similar and the differences are illustrated in Figures S4–S6 (see the Supplementary Materials).

Analyzing the spectra obtained for the four cellulosic derivative–polypropylene hollow fiber composite membranes (Figure 6), the following wave numbers were chosen to create the 2D maps:

- 3386 cm^−1^ (–O–H stretching vibration from cellulose);
- 2950 cm^−1^ (C–H stretching vibration from PP and cellulose);
- 1639 cm^−1^ (C–O stretching vibration from cellulose);
- 1170 cm^−1^ (C–C stretching vibration from PP and cellulose).

The chosen wavelengths give the 2D FTIR maps a high relevance because they are in the range of the minimum transmittance value of the superimposed FTIR spectra (Figure 6b).

**Figure 7:**
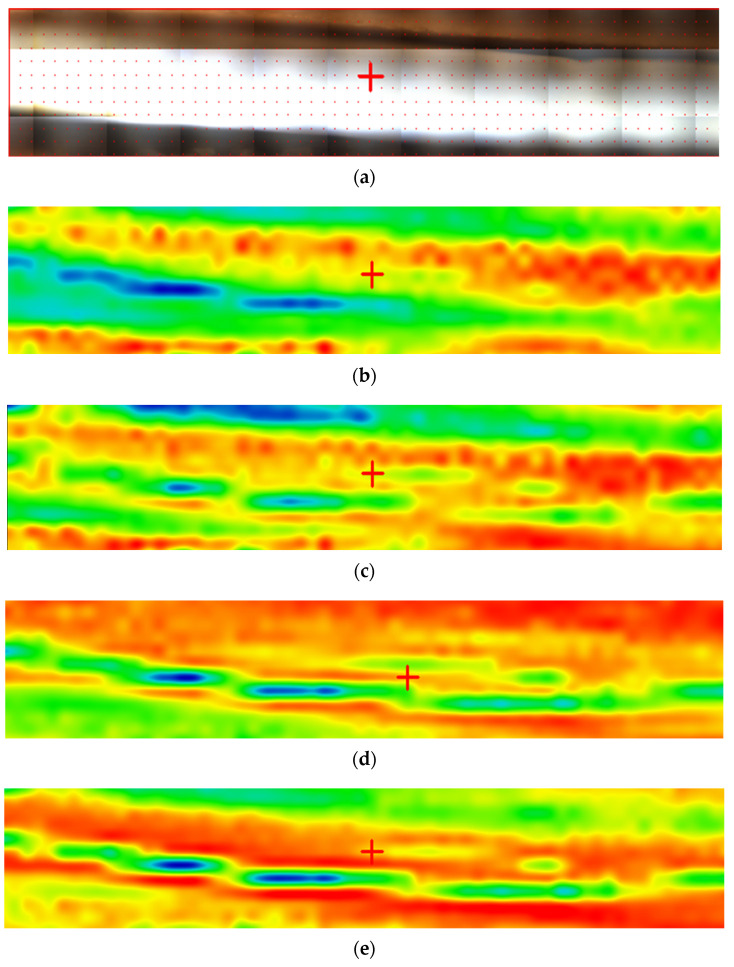

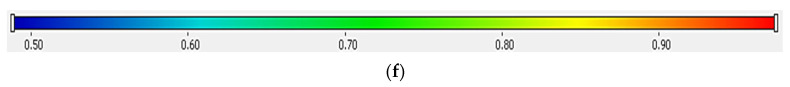
FTIR 2D maps for sodium carboxymethyl–cellulose–polypropylene hollow fiber membrane: (**a**) video image; (**b**) 2D image at wavenumber 3386 cm^−1^; (**c**) 2D image at wavenumber 2950 cm^−1^; (**d**) 2D image at wavenumber 1639 cm^−1^; and (**e**) 2D image at wavenumber 1170 cm^−1^; (**f**) color scale.

Figure 7 shows the 2D maps for the composite membrane P1, and the Supplementary Materials provides the information for the membranes P2, P3, and P4 (see Figures S7–S9 in the Supplementary Materials).

The 2D maps show a varied distribution of the cellulosic derivative on the membrane surface: from 50% (in blue) in the center of the image center to 90% (orange to red) in the sides (Figure 7b–e). The way the colors are distributed suggest that the cellulosic derivative enters the membrane pores in the area where they are more abundant. It is worth noting that in all 2D maps the coloring of the areas is close. Similar information is provided by the 2D maps of the composite membranes P2, P3, and P4.

**Figure 8:**
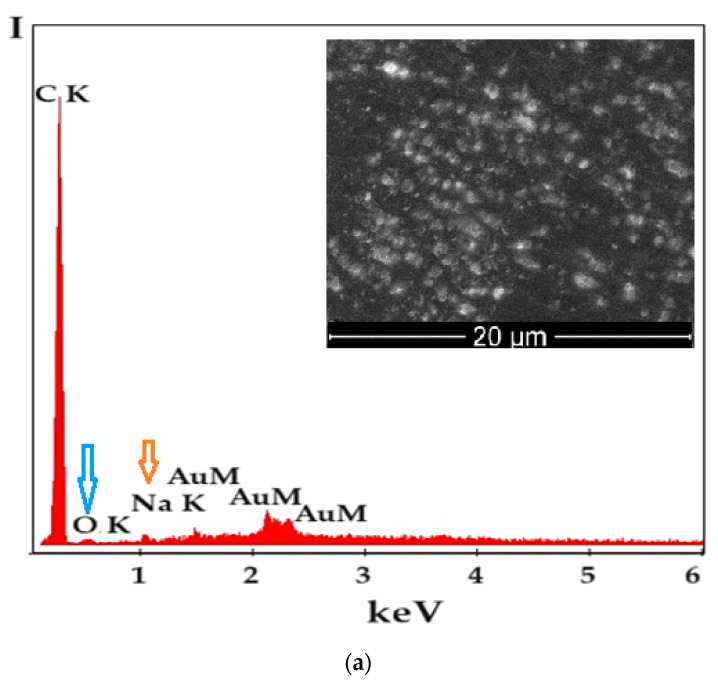

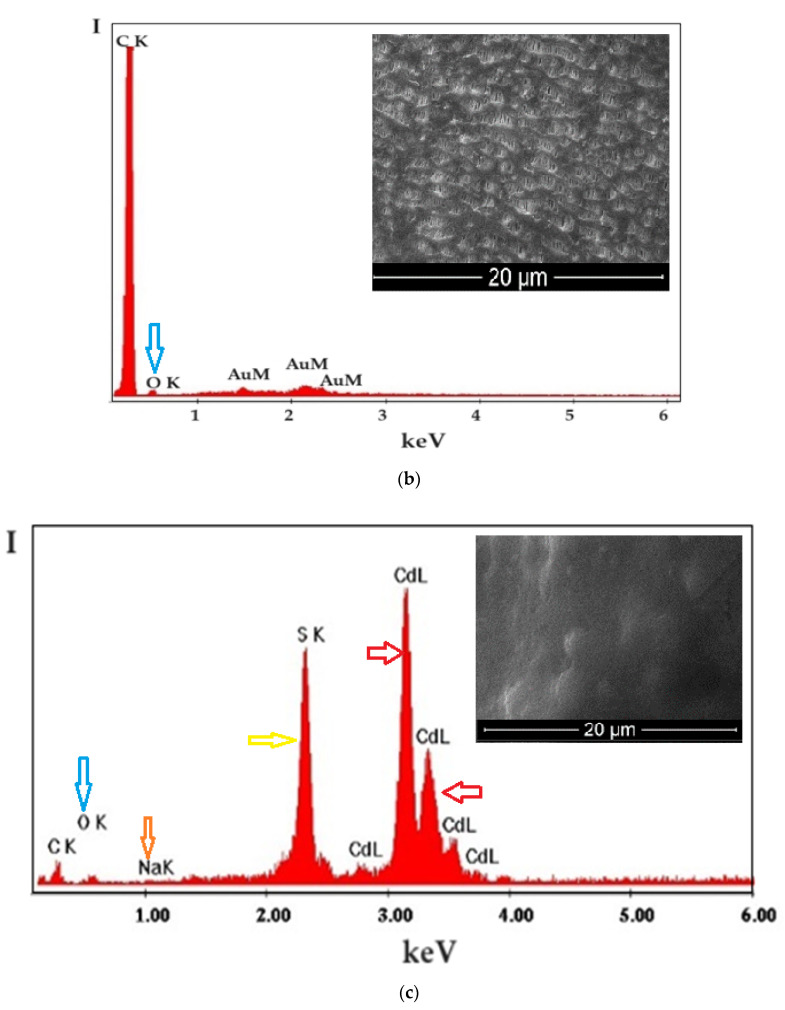
Structural characterization of composite membranes by EDAX for membranes: (**a**) P1; (**b**) P2 before hydrogen sulfide retention process; and (**c**) P5 after retention of hydrogen sulfide as cadmium sulfide.

The structural characterization of the composite membranes (P1, P3, and P5) was completed with analysis through Energy Dispersive X-ray Spectroscopy EDAX (Figure 8).

The EDAX spectrum for the composite membrane sodium carboxymethyl–cellulose–polypropylene hollow fiber membrane (P1) (Figure 8a) highlights the composition in sodium (orange arrow) and oxygen (blue arrow).

The EDAX spectrum for the methyl 2 hydroxyethyl–cellulose (MHEC)–polypropylene hollow fiber membrane (P2) composite membrane (Figure 8b) shows that carbon and oxygen (blue arrow) elements are present.

The EDAX spectrum for the composite membrane sodium carboxymethyl–cellulose–polypropylene hollow fiber membrane, after retention of hydrogen sulfide as cadmium sulfide (P5), indicates the presence of sodium (orange arrow), sulfur (yellow arrow), and cadmium (red arrows) (Figure 8c). The strong presence of cadmium and sulfur shows that the cadmium sulfide almost completely covers the composite membrane, which is also confirmed in Figure 5.

For each of the presented spectra, the SEM image of the membrane area examined through DAX is indicated in the upper right, and the constituent elements (except for carbon) are highlighted with colored arrows in the spectra.

From the EDAX spectra (Figure 8a,b), traces of the gold film covering the composite polymer membranes P1 and P2 are visible. The P5 membrane formed after the hydrogen sulfide retention process as cadmium sulfide did not require gold coating (Figure 8c).

### 3.2. Thermal Characterization

The thermal analysis TG-DSC for the precursors was performed with a Netzsch STA 449C Jupiter apparatus. The cut samples (~4 mg) were placed in an open crucible made of alumina and heated with 10 K/min from room temperature up to 650 °C, under the flow of 50 mL/min dried air. An empty alumina crucible was used as reference.

**Figure 9:**
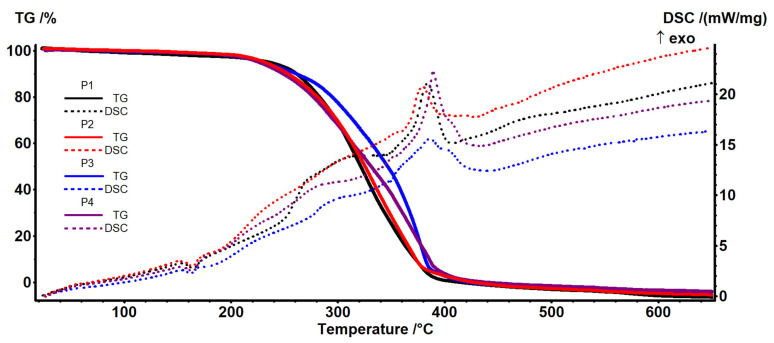
Thermal diagrams for the four composite membranes (P1, P2, P3, and P4).

**Figure 10:**
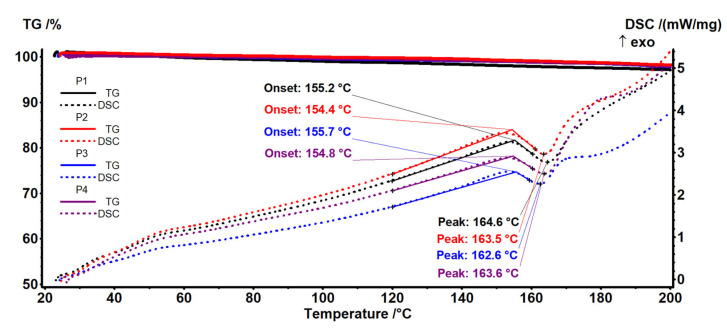
Thermal diagrams for the four composite membranes (P1, P2, P3, and P4) (onset diagram).

Samples P1–P4 (Figure 9 and Figure 10) have a similar thermal behavior due to the presence of polypropylene fibers in their composition. The details necessary for the interpretation of the thermal diagrams are provided by the additional Figures S10–S13 (see Supplementary Materials), being presented next. The samples are losing around ~2.1–2.9% up to 200 °C due to solvent elimination and the degradation of less stable terminal moieties. The endothermic effect recorded on the DSC curve in this temperature interval is caused by the melting of the polypropylene in the range 154–156 °C (Figure 9).

After 200 °C, degradation by the fragmentation of polymer backbone and oxidation of the fragments is recorded (Figure 9 and Figure 10). The overall DSC effect is exothermic, indicating the dominance of oxidation reactions over the fragmentation of the polymer chain.

Up to 415 °C, the samples are completely degraded, with the exothermic effect from 380 to 390 °C being attributed to the burning of the residual carbonaceous mass.

The principal numerical data from the thermal analysis are presented in Table 2.

**Figure 11:**
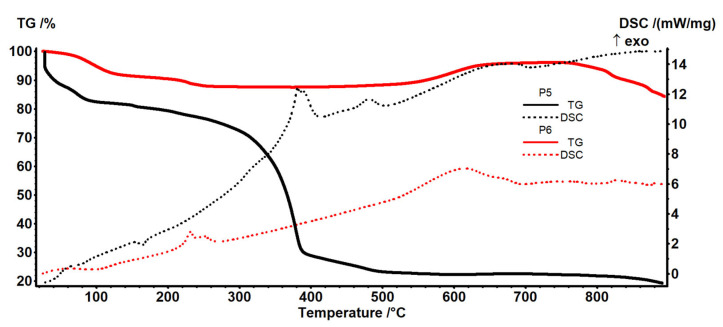
The TG-DSC curves for the P5 sample (membrane P1 after retention of hydrogen sulfide as CdS).

The details of the interpretation of Figure 11 are depicted in the thermal diagrams in the Supplementary Materials (Figure S14a,b) and presented below. After the process, the sodium carboxymethyl–cellulose–polypropylene hollow fiber membrane (P5) (Figure 11) wetted with the test solution was losing the present solvent up to 200 °C. In this interval, the melting of the fibers was observable at 154.7 °C [59,68], with a melting enthalpy of 25.01 J/g.

The degradation of the polymer backbone starts after 200 °C by fragmentation and oxidation, as previously reported in [57,69]. The oxidation process of the organic fragments is associated with the exothermic effect at 381.7 °C, while the burning of the residual carbonaceous mass is assigned to the exothermic peak at 482.0 °C.

A small exothermic effect at 683.2 °C is associated with a mass increase of 0.32% and can be attributed to the oxidation of CdS to CdSO_4_ and related species, as proven by CdS thermal analysis [70,71].

**Figure 12:**
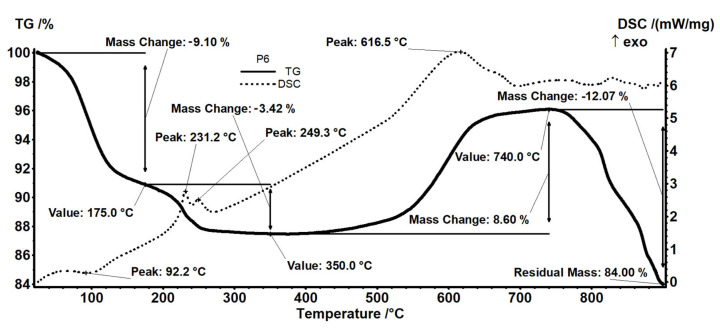
The thermal analysis (TG and DSC curves) for sample P6 (CdS from retention of hydrogen sulfide on membrane P1).

The P6 sample (CdS obtained in the hydrogen sulfide retention process on the membrane P1) (see Figure 12) exhibits a mass loss of 9.10% up to 175 °C, which can be assigned to residual humidity/crystallization water. The process is accompanied by a weak endothermic process on the DSC curve, with the minimum at 92.2 °C. The sample is losing 3.42% of its initial mass between 175 and 350 °C in an oxidative event, as indicated by the double exothermic peaks on the DSC curve, at 231.1 °C and 249.3 °C. This can imply the simultaneous elimination of water and oxidation of CdS or the obtaining of CdO by Reaction (5):

2CdS + 3O_2_ = 2CdO + 2SO_2_ (5)

as reported before [70].

The mass increase of 8.60% in the interval 350–740 °C indicates a further oxidation to CdSO_4_ or other species (Cd_5_S_3_O_6_; Cd(S_2_O_7_)) [70]. The oxidation process is accompanied by a strong and broad exothermic effect on the DSC curve, with maximum at 616.5 °C.

After 740 °C, the compound starts to decompose, losing 12.07% of its mass up to 900 °C, with the literature proposing some reactions like (6) and (7) [70,71].

2CdS + 3O_2_ = 2CdO + 2SO_2_ (6)

CdS + CdSO_4_ = 2CdO + 2SO_2_ (7)

Thermal data show that sodium carboxymethyl–cellulose–polypropylene hollow fiber membrane (P5) after processing can be used by heating it up to 200 °C, when both the polymer material and the cadmium sulfide retain their properties, but that the polymer material dehydrates and melts, and nanometric cadmium sulfide is dehydrated.

The polymeric material containing nanometric cadmium sulfide could be used in the manufacturing process of reflective materials, including road ones.

### 3.3. Performance Processes for Hydrogen Sulfide Recuperative Separation

In this part of the work, the results of hydrogen sulfide retention from closed rooms, rooms in residential houses, or hotels are presented.

The study parameters were chosen as follows:

- Volume of polluted air of 5.0 m^3^;
- Surface of the composite membrane of 0.1 m^2^;
- Composite membrane cellulosic derivative–polypropylene hollow fiber:
  ○ Sodium carboxymethyl–cellulose–polypropylene hollow fiber (P1);
  ○ Cellulose acetate–polypropylene hollow fiber (P2);
  ○ Methyl 2–hydroxyethyl–cellulose–polypropylene hollow fiber (P3);
  ○ Hydroxyethyl–cellulose–polypropylene hollow fiber (P4).
- Hydrogen sulfide concentrations of 20 ppm, 40 ppm, and 60 ppm;
- Hydrogen sulfide flow rates: 50 L/min, 100 L/min, and 150 L/min;
- pH of the cadmium nitrate receiving phase solution: 0, 2, 4, and 6.

#### 3.3.1. Influence of the Nature of the Composite Membrane on the Hydrogen Sulfide Pertraction Efficiency

**Figure 13:**
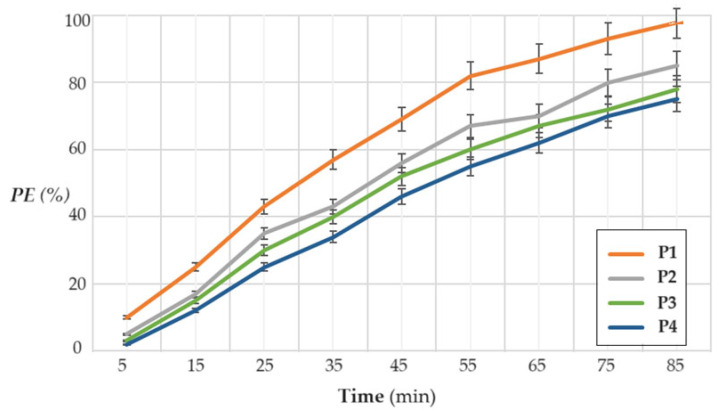
The variation in pertraction efficiency (PE%) as a function of time for the following membranes: sodium carboxymethyl–cellulose–polypropylene hollow fiber (P1), cellulose acetate–polypropylene hollow fiber (P2), methyl 2–hydroxyethyl–cellulose–polypropylene hollow fiber (P3), and 2–hydroxyethyl–cellulose–polypropylene hollow fiber (P4).

Probably operating in the most unfavorable working conditions—regarding the concentration of hydrogen sulfide (60 ppm), flow-rate (150 L/min), a maximum operating time of 85 min, and a pH = 6—the sodium carboxymethyl–cellulose–polypropylene hollow fiber (P1), cellulose acetate–polypropylene hollow fiber (P2), methyl 2–hydroxyethyl–cellulose–polypropylene hollow fiber (P3), and hydroxyethyl–cellulose–polypropylene hollow fiber (P4) membranes have the behavior indicated in Figure 13.

The pertraction efficiency over the entire time interval decreases in the following order: sodium carboxymethyl–cellulose–polypropylene hollow fiber membrane (P1) >> cellulose acetate–polypropylene hollow fiber membrane (P2) > methyl 2–hydroxyethyl–cellulose–polypropylene hollow fiber membrane (P3) > 2–hydroxyethyl–cellulose–polypropylene hollow fiber membrane (P4).

The results obtained for the P1 membrane are, most likely, superior to the other three as a result of different interactions with hydrogen sulfide, although it has a very small dipole moment (Table 3). Thus, sodium carboxymethyl–cellulose–polypropylene hollow fiber membrane (P1) interacts with hydrogen sulfide through the ion–dipole bond with the carboxylate groups, and with the hydroxyl group and etheric oxygen through the dipole–dipole bonds. Cellulose acetate–polypropylene hollow fiber membrane (P2) establishes dipole–dipole bonds with ester and hydroxyl groups, as well as with ether oxygen. Methyl 2–hydroxyethyl–cellulose–polypropylene hollow fiber membrane (P3) has dipole–dipole interactions with the hydroxyl group and ether oxygen, while 2–hydroxyethyl–cellulose–polypropylene hollow fiber membrane (P4) interacts dipole–dipole only with oxygen atoms from the ether groups.

Hydrogen sulfide also has hydrophobic bonds with all the studied membranes.

On the other hand, in the proposed system, an essential aspect is the hydration of the membrane material, which consists of hydrophilic polymers. The transfer of hydrogen sulfide from the gaseous source phase (impure air) is carried out through the hydration water, of the membrane, to the receiving aqueous phase (aqueous cadmium nitrate solution).

All the polymers used in the study are specifically hydrophilic, and their hydration water reacts with the hydrogen sulfide in the source phase, forming SH^−^ and S^2−^ ions, according to Reactions (8) and (9):

H_2_S + HOH ⇌ HS^−^ + H_3_O^+^ (8)

HS^−^ + HOH ⇌ S^2−^ + H_3_O^+^ (9)

The chemical species present in the hydration water (H_2_S molecules, SH^−^ and S^2−^ ions) diffuse from the membrane towards the receiving phase where they are fixed as cadmium sulfide.

In conclusion, the mechanism for separating hydrogen sulfide (from impure air) in the proposed system is that of solubilization–diffusion through the hydrated membrane, followed by the precipitation reaction of cadmium sulfide in the receiving phase.

Following all these interactions, the pertraction efficiency decreases in the following series: P1 >> P2 > P3 > P4.

This observation is highly correlated with the minimum transmittance value, due to the hydrogen bonds, in FTIR spectra of P1, P2, P3, and P4 membranes (Figure 6b) in the 3200–3600 cm^−1^ range.

Because the membrane sodium carboxymethyl–cellulose–polypropylene hollow fiber (P1) has the best pertraction efficiency, it will be chosen for the continuation of the study by varying the other parameters.

#### 3.3.2. Influence of Hydrogen Sulfide Concentration on Hydrogen Sulfide Pertraction Efficiency

The concentration of hydrogen sulfide in the polluted air is the determining parameter for bringing the polluted air to bearable values and affecting health as little as possible.

In the conditions of some areas with mofettas or extinct volcanoes (e.g., Covasna-Harghita area, Romania) the concentration of hydrogen sulfide in homes near the generating sources is between 1.0 ppm and 50.0 ppm.

**Figure 14:**
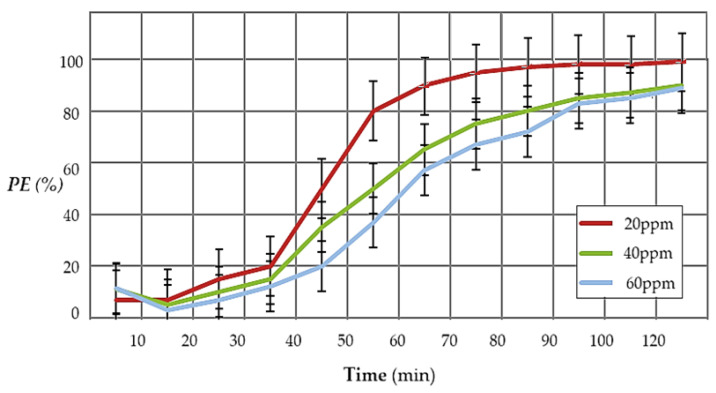
The variation in the pertraction efficiency depending on the operating time, at hydrogen sulfide concentrations of 20, 40, and 60 ppm, for the membrane sodium carboxymethyl–cellulose–polypropylene hollow fiber (P1).

For the study of the pertraction variation (PE%), depending on the operating time using the composite membrane (P1) (Figure 14), three concentrations of hydrogen sulfide were chosen, which are at the limit of respiratory tract irritation (20 ppm, 40 ppm, and 60 ppm). The membrane used was sodium carboxymethyl–cellulose–polypropylene hollow fiber (P1), the working flow was 150 L/min, the pH of the cadmium nitrate solution equal to 2, and the cadmium nitrate concentration 10^−1^ mol/L.

The obtained results show that the efficiency of the separation increases with the decrease in the concentration of hydrogen sulfide in the polluted air. Also, the results suggest operating in two or three permeation modules in series, or recirculating air through the module until cadmium is depleted from the cadmium nitrate receptor phase.

#### 3.3.3. The Influence of the Flow Rate of the Air Polluted with Hydrogen Sulfide on the Efficiency of Hydrogen Sulfide Pertraction

The flow rate of the hydrogen sulfide polluted air is conditioned by the volume of the premises subjected to experiment and the time in which we want to bring the concentration of hydrogen sulfide to bearable olfactory limits.

**Figure 15:**
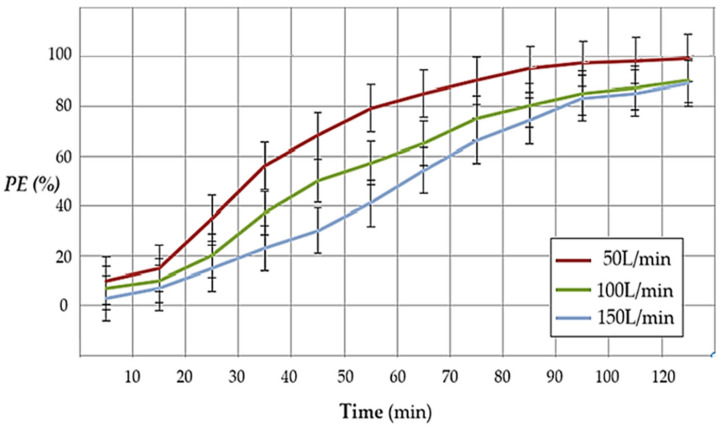
The variation in hydrogen sulfide pertraction efficiency as a function of time and the flow rate of polluted air for the composite membrane P1, with an air volume of 5 m^3^, a concentration of 60 ppm, and pH = 2 in the receiving phase.

In Figure 15, for a concentration of 60 ppm, pH = 2 in the receiving phase, a volume of air in the considered enclosure (premise) of 5.0 m^3^, and the composite membrane P1, it is observed that a small flow favors the efficiency of hydrogen sulfide pertraction and that the operating time reaches two hours for the flow rate of 50 L/min and over two hours at the flow rates of 100 L/min and 150 L/min.

The work flux is also conditioned by the need to pass the entire air volume of the considered room through the pertraction module at least once.

The hydrogen sulfide flux rates (Table 4), for membrane P1, fall between 0.25 × 10^−7^ mol·m^−2^·s^−1^ for the values of Q_H2S_ = 150 L/min, C_H2S_ = 20 ppm, and pH = 2 and 0.67 × 10^−7^ mol·m^−2^·s^−1^ for the values of Q_H2S_ = 50 L/min, C_H2S_ = 60 ppm, and pH = 2.

The flow values highlighted in Table 4 (in bold) show that, under the same working conditions, the results are repeatable and reproducible.

#### 3.3.4. The Influence of the pH of the Cadmium Nitrate Solution on the Hydrogen Sulfide Pertraction Efficiency

The pH of the receiving phase of cadmium nitrate with a concentration of 0.1 mol/L influences the efficiency of hydrogen sulfide pertraction (Table 5). The conditions in which data were obtained are as follows: a composite membrane (P1) surface of 0.1 m^2^, a hydrogen sulfide concentration of 20 ppm, a work flow of 50 L/min, an operating time of 2 h, and the volume of treated polluted air as 5 m^3^.

The results show that the optimal operating pH range is between 2 and 4. At low pH, cadmium sulfide does not reach the precipitation pH, and at high pH it precipitates simultaneously with cadmium sulfide and cadmium carbonate coming from cadmium ions and the carbon dioxide from polluted air. A further increase in pH is undesirable because the hydroxyl ions that precipitate the cadmium hydroxide will enter the competition.

**Figure 16:**
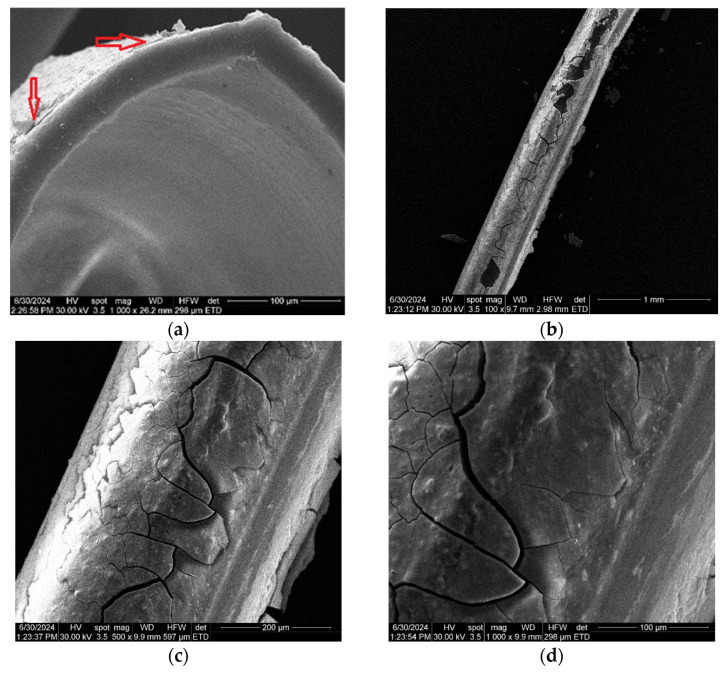
Scanning electron microscopy (SEM) for membrane P1 coated with cadmium sulfide following hydrogen sulfide pertraction in a cadmium nitrate solution: (**a**) the membrane section after the hydrogen sulfide recovery process as CdS; (**b**) the membrane view at a 100× resolution; (**c**) the membrane view at a 500× resolution; and (**d**) the membrane view at a 1000× resolution. The layer of cadmium sulfide is indicated by red arrows.

Following the pertraction experiments of hydrogen sulfide from the polluted air, through sodium carboxymethyl–cellulose–polypropylene hollow fiber membrane (P1), with a cadmium nitrate receptor phase at pH 2, a working flow rate of 50 L/min, at a working time of 2 h, the membrane covered with cadmium sulfide (P5) (Figure 16) is obtained, suitable for thermal treatment (melting) and obtaining a reflective material.

Figure 16a shows the SEM image of the P5 membrane section, in which the layer of cadmium sulfide can be observed, indicated by the red arrows. Figure 16b–d highlight aspects regarding the adhesion and microcracks specific to the drying of this layer under the vacuum conditions specific to the examination of the samples under high vacuum. The way the cadmium sulfide layer is presented (Figure 8a–c) can ensure the melting of the composite membranes together with the cadmium sulfide to obtain a reflective material. Obtaining the proposed reflective material is supported by the nanometric dimensions of the deposited cadmium sulfide (Figure 5e).

### 3.4. Proposal of a System for Separating Hydrogen Sulfide as Cadmium Sulfide at a Low Concentration

**Figure 17:**
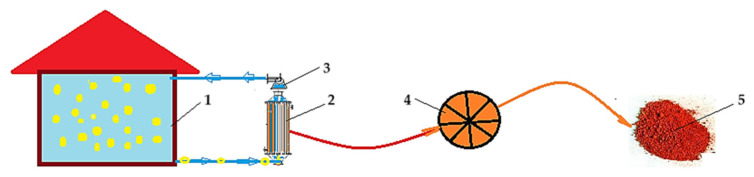
A schematic presentation of the proposed system for the retention of hydrogen sulfide, in low concentrations, as cadmium sulfide, through cellulosic derivative–polypropylene hollow fiber composite membranes: 1—enclosure with impure air, 2—pertraction module with composite membrane P1, 3—fan, 4—mill, 5—obtained reflective material.

The system proposed for the retention of hydrogen sulfide as cadmium sulfide, through composite cellulosic derivative–polypropylene hollow fiber membranes, is shown schematically in Figure 17.

In the considered premise (1), there is 50 m^3^ of impure air with a concentration of 60 ppm hydrogen sulfide. The impure air is transmitted through the composite membranes of the polypropylene pertraction module, with a P1 membrane surface of 1 m^2^ (2), and then is recirculated, with a flow rate of 150 L/min, with the help of the fan (3). Outside the membranes in the separation module (2) there are 10 L of cadmium nitrate solution with a concentration 1 mol/L. The operating time of the installation is 360 min, and the pertraction efficiency is 95%. After separation, the polypropylene module containing sodium carboxymethyl–cellulose propylene hollow fiber composite membranes on which cadmium sulfide is deposited is detached and inserted into the mill (4). From the mill (4), the reflective material (5) cadmium sulfide–polypropylene with traces of cellulosic derivative is obtained.

Under the indicated conditions, the impure air reaches a concentration of 5 ± 1 ppm. To further reduce the concentration, the operating time of the separation process can be increased.

A pertraction module, in a separation system like the one in Figure 17, can work efficiently for 30 days, after which it must be replaced, because the cadmium nitrate solution runs out.

## 4. Conclusions

Treating polluted air with hydrogen sulfide is an important problem for the residential premises in areas with mofettas or extinct volcanoes.

This paper presents the results of hydrogen sulfide removal from an enclosure with a defined volume using cellulosic derivative–polypropylene hollow fiber composite membranes. All membranes obtained were characterized by SEM, EDAX, FTIR, 2D FTIR maps, and thermal analysis (TG, DSC). Among the four prepared membranes—sodium carboxymethyl–cellulose–polypropylene hollow fiber (P1), cellulose acetate–polypropylene hollow fiber (P2), methyl 2–hydroxyethyl–cellulose–polypropylene hollow fiber (P3), and 2–hydroxyethyl–cellulose–polypropylene hollow fiber (P4)—the best results of hydrogen sulfide pertraction and capture in a receiving phase of cadmium nitrate solution were obtained with the composite membrane P1.

The mechanism for separating hydrogen sulfide (from impure air) in the proposed system is that of solubilization—diffusion through the hydrated membrane, followed by the precipitation reaction of cadmium sulfide, in the receiving phase.

The hydrogen sulfide flux rates, for membrane P1, fall between 0.25 × 10^−7^ mol·m^−2^·s^−1^ for the values of Q_H2S_ = 150 L/min, C_H2S_ = 20 ppm, and pH = 2 and 0.67 × 10^−7^ mol·m^−2^·s^−1^ for the values of Q_H2S_ = 50 L/min, C_H2S_ = 60 ppm, and pH = 2. These fluxes are below those reported in the literature for high concentrations of hydrogen sulfide in various gasses.

The optimal operating conditions, obtained with the working parameters presented in the paper, are as follows: a flow rate of 50 L/min of air polluted with 20 ppm hydrogen sulfide, and a pH between 2 and 4 for the receiving phase of cadmium nitrate with a concentration of 0.1 mol/L.

The membrane covered with cadmium sulfide, stable up to 200 °C, is recommended for heat treatment (melting) and obtaining reflective material for road markings.

The paper proposes a simple air purification system containing hydrogen sulfide using a cellulosic derivative–polypropylene hollow fiber composite membrane module.

## Figures and Tables

**Table 1:** The characteristics of the tested cellulosic derivatives and the obtained membranes.

Cellulosic Derivatives (Cell-D)	Chemical Formula	Molar Weight	Polypropylene Hollow Fiber–Cellulosic Derivative Membrane Symbol
sodium carboxymethyl–cellulose (NaCMC)	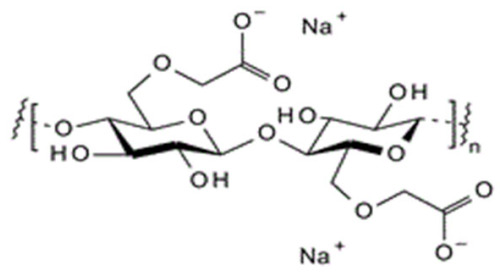	90,000	P1
cellulose acetate (CA)	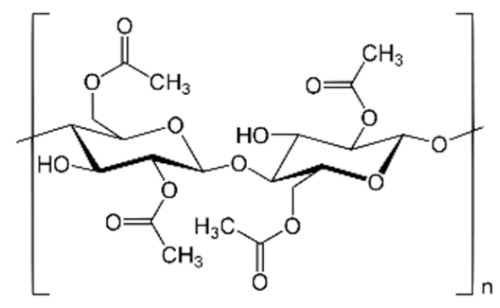	50,000	P2
methyl 2–hydroxyethyl–cellulose (MHEC)	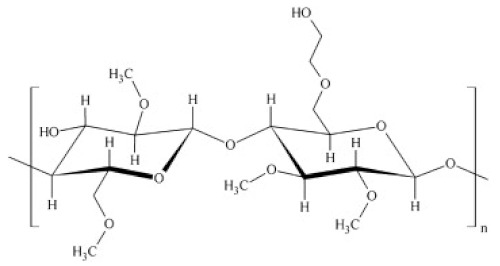	not applicable	P3
hydroxyethyl–cellulose (HEC)	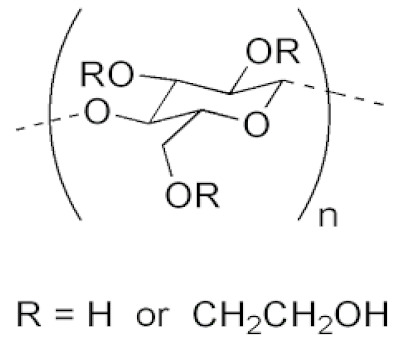	90,000	P4

**Table 2:** Principal numerical data from thermal analysis for P1–P4 samples.

Sample	T5% (°C)	T10% (°C)	T50% (°C)	Mass Loss % RT–200 °C	Melting Onset (°C)	Melting Peak (°C)	Exothermic Effect (°C)
P1	235	264	322	2.88	155.2	164.6	384.1
P2	230	252	325	2.12	154.4	163.5	379.5
P3	227	260	346	2.48	155.7	162.6	385.8
P4	224	247	331	2.50	154.8	163.6	389.3

**Table 3:** Interaction groups of membranes with hydrogen sulfide.

Membrane	P1	P2	P3	P4
Functional groups interacting with H_2_S	−COO^−^; −OH; −O−	−COOR; −OH; −O−	−OH; −O−	−O−

**Table 4:** Variation in hydrogen sulfide flows depending on variable parameters.

Constant Parameters	Membrane (P1) Q_H2S_ = 150 L/min; pH = 2			Membrane (P1) C_H2S_ = 60 ppm; pH = 2		
Variable Parameters	C_H2S_ (ppm)			Q (L/min)		
	20	40	60	50	100	150
Flux·10^7^ (mol m^−2^ s^−1^)	0.25	0.32	**0.42**	0.67	0.500	**0.42**

**Table 5:** The dependence of hydrogen sulfide pertraction efficiency on the pH of the receiving phase.

pH of Receiving Phase	0	2	4	6
PE (%)	86.3	98.3	97.2	89.6

## Data Availability

The original contributions presented in the study are included in the article/Supplementary Materials; further inquiries can be directed to the corresponding authors.

## Supplementary Materials

The following supporting information can be downloaded at https://www.mdpi.com/article/10.3390/ma17174437/s1, Figure S1: scanning electron microscopy for cellulose acetate–polypropylene hollow fiber membrane (P2); Figure S2: scanning electron microscopy for methyl 2–hydroxyethyl–cellulose (MHEC)–polypropylene hollow fiber membrane (P3); Figure S3: scanning electron microscopy for hydroxyethyl–cellulose (HEC)–polypropylene hollow fiber membrane (P4); Figure S4: Fourier Transform Infra-Red (FTIR) spectra for P2 composite membrane; Figure S5: Fourier Transform Infra-Red (FTIR) spectra for P3 composite membrane; Figure S6: Fourier Transform Infra-Red (FTIR) spectra for P4 composite membrane; Figure S7: FTIR 2D maps for cellulose acetate–polypropylene hollow fiber membrane; Figure S8: FTIR 2D maps for methyl 2–hydroxyethyl–cellulose–polypropylene hollow fiber membrane; Figure S9: FTIR 2D maps for hydroxyethyl–cellulose–polypropylene hollow fiber membrane; Figure S10: detail of thermal diagrams for P1; Figure S11: detail of thermal diagrams for P2; Figure S12: detail of thermal diagrams for P3; Figure S13: detail of thermal diagrams for P4; Figure S14: zoom-in detail for Figure 11, in the case of P5; Figure S15: detail of thermal diagrams for P5.
