# Supplementary material for: Reduction in Olfactory Discomfort in Inhabited Premises from Areas with Mofettas through Cellulosic Derivative–Polypropylene Hollow Fiber Composite Membranes
Source: Materials (Basel). 2024 Sep 9;17(17):4437. doi: 10.3390/ma17174437 (PMC11396629; doi:10.3390/ma17174437)
Supplement: Supplementary file 1 [file materials-17-04437-s001.zip › materials-3140645-supplementary.pdf]

Article

Reduction of olfactory discomfort in inhabited premises from areas with mofettas through cellulosic derivative–polypropylene hollow fiber composite membranes

Paul Constantin Albu [1], Andreia Pîrțac [2], Ludmila Motelica [3], Aurelia Cristina Nechifor [2], Geani Teodor Man [2,4], Alexandra Raluca Grosu [2], Szidonia-Katalin Tanczos [5], Vlad-Alexandru Grosu [6,] *, Gheorghe Nechifor [3,] *

[1] Radioisotopes and Radiation Metrology Department (DRMR), IFIN Horia Hulubei, Măgurele 023465, Romania; paulalbu@gmail.com (P.C.A.);

[2] Analytical Chemistry and Environmental Engineering Department, National University of Science and Technology POLITEHNICA Bucharest, Bucharest 011061, Romania; man_geani@yahoo.com (G.T.M.); andreia.pascu@yahoo.ro (A.P.); aureliacristinanechifor@gmail.com (A.C.N.); alexandra.grosu@upb.ro (A.R.G.); gheorghe.nechifor@upb.ro (G.N.);

[3] National Research Center for Micro and Nanomaterials, Department of Science and Engineering of Nanomaterials and oxide materials, National University of Science and Technology POLITEHNICA Bucharest, Bucharest 060042, Romania; ludmila.motelica@upb.ro (L.M.);

[4] National Research and Development Institute for Cryogenics and Isotopic Technologies–ICSI, Râmnicu-Vâlcea 240050, Romania; geani.man@icsi.ro (G.T.M.);

[5] Department of Bioengineering, University Sapientia of Miercurea-Ciuc, Miercurea-Ciuc 500104, Romania; tczszidonia@yahoo.com (S.-K.T.);

[6] Department of Electronic Technology and Reliability, Faculty of Electronics, Telecommunications and Information Technology, National University of Science and Technology POLITEHNICA Bucharest, Bucharest 061071, Romania; vlad.grosu@upb.ro (V.-A.G.);

* Correspondence: gheorghe.nechifor@upb.ro; vlad.grosu@upb.ro

Abstract: Hydrogen sulfide is present in active or extinct volcanic areas (mofettas). The habitable premises in these areas are affected by the presence of hydrogen sulfide, which, even in low concentrations, gives off a bad to unbearable smell. If the living spaces considered are closed enclosures, then a system can be designed to reduce the concentration of hydrogen sulfide. This paper presents a membrane way to reduce the hydrogen sulfide concentration to acceptable limits using a cellulosic derivative-propylene hollow fiber based composite membrane module. The cellulosic derivatives considered were: carboxymethyl–cellulose (NaCMC), P1; cellulose acetate (CA), P2; methyl 2–hydroxyethyl–cellulose (MHEC), P3; and 2–hydroxyethyl–cellulose (HEC), P4. In the permeation module, hydrogen sulfide is captured with a solution of cadmium that forms cadmium sulfide, usable as a luminescent substance. The composite membranes were characterized by SEM, EDAX, FTIR, FTIR 2D maps, thermal analysis (TG and DSC) and from the perspective of hydrogen sulfide air removal performance. To determine the process performances, the variables were: the nature of the cellulosic derivative–polypropylene hollow fiber composite membrane, the concentration of hydrogen sulfide in the polluted air, the flow rate of polluted air, and the pH of the cadmium nitrate solution. Pertraction efficiency was maximum for the sodium carboxymethyl–cellulose (NaCMC)-polypropylene hollow fiber membrane, a hydrogen sulfide concentration in the polluted air of 20 ppm, polluted air flow rate of 50 L/min, and a pH of 2 and 4. The hydrogen sulfide flow rates, for membrane P1, fall between 0.25×10^{-7} $mol\cdot m^{-2}\cdot s^{-1}$ for the values of Q_{H2S} = 150 L/min, C_{H2S} = 20 ppm, pH=2, and 0.67×10^{-7} $mol\cdot m^{-2}\cdot s^{-1}$ for the values of Q_{H2S} = 50 L/min, C_{H2S} = 60 ppm and pH=2. The paper proposes a simple air purification system containing hydrogen sulfide, using a module with composite cellulosic derivative–polypropylene hollow fiber membranes.

Keywords: hydrogen sulfide separation, polluted air, composite membranes, sodium carboxymethyl–cellulose, cellulose acetate, methyl 2–hydroxyethyl–cellulose, hydroxyethyl–cellulose

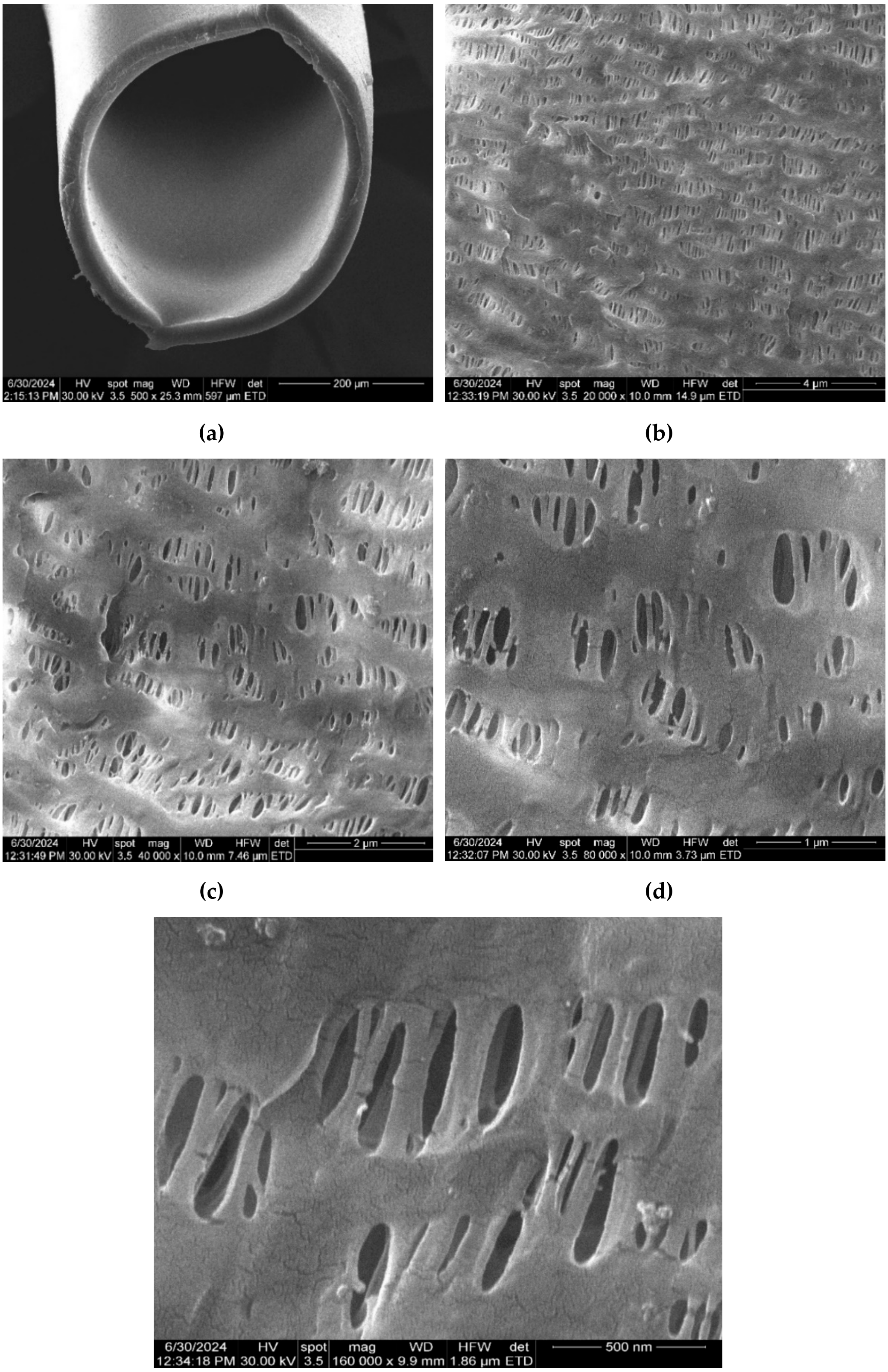

(e)

Figure S1. Scanning electron microscopy for cellulose acetate–polypropylene hollow fiber membrane (P2): (**a**) composite membrane section; (**b**) membrane surface at 20,000× magnification; (**c**) membrane surface at 40,000× magnification; (**d**) membrane surface at 80,000× magnification; and (**e**) membrane surface at 160,000× magnification.

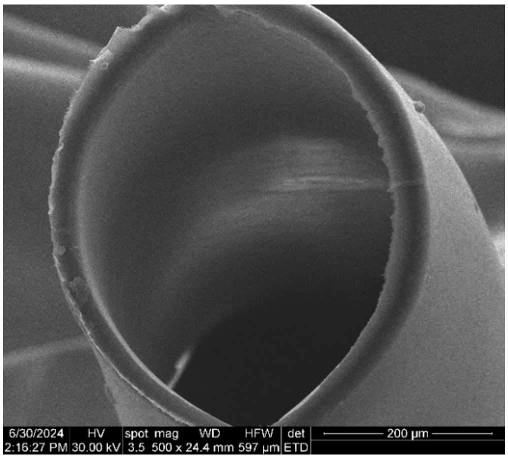

(a)

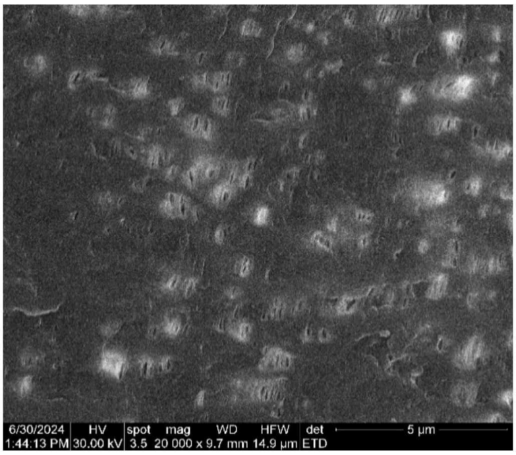

(b)

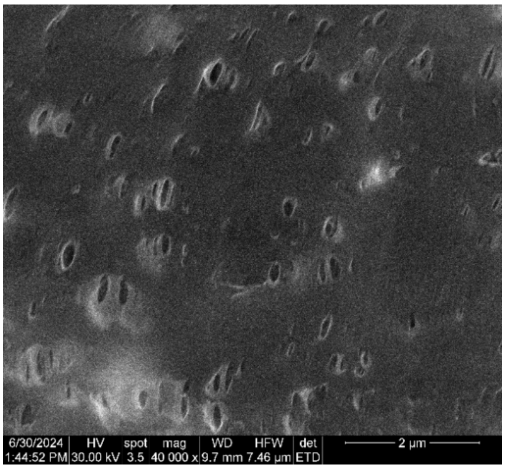

(c)

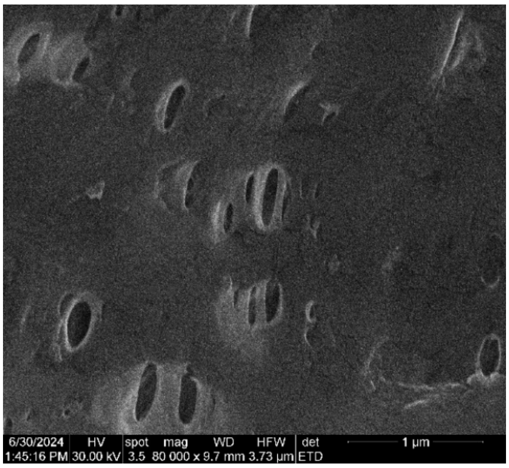

(d)

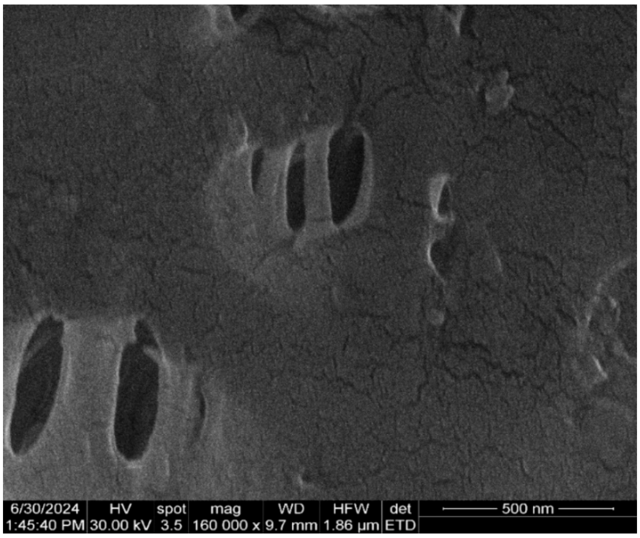

(e)

Figure S2. Scanning electron microscopy for methyl 2–hydroxyethyl–cellulose (MHEC)–polypropylene hollow fiber membrane (P3): (**a**) composite membrane section; (**b**) membrane surface at 20,000× magnification; (**c**) membrane surface at 40,000× magnification; (**d**) membrane surface at 80,000× magnification; and (**e**) membrane surface at 160,000× magnification.

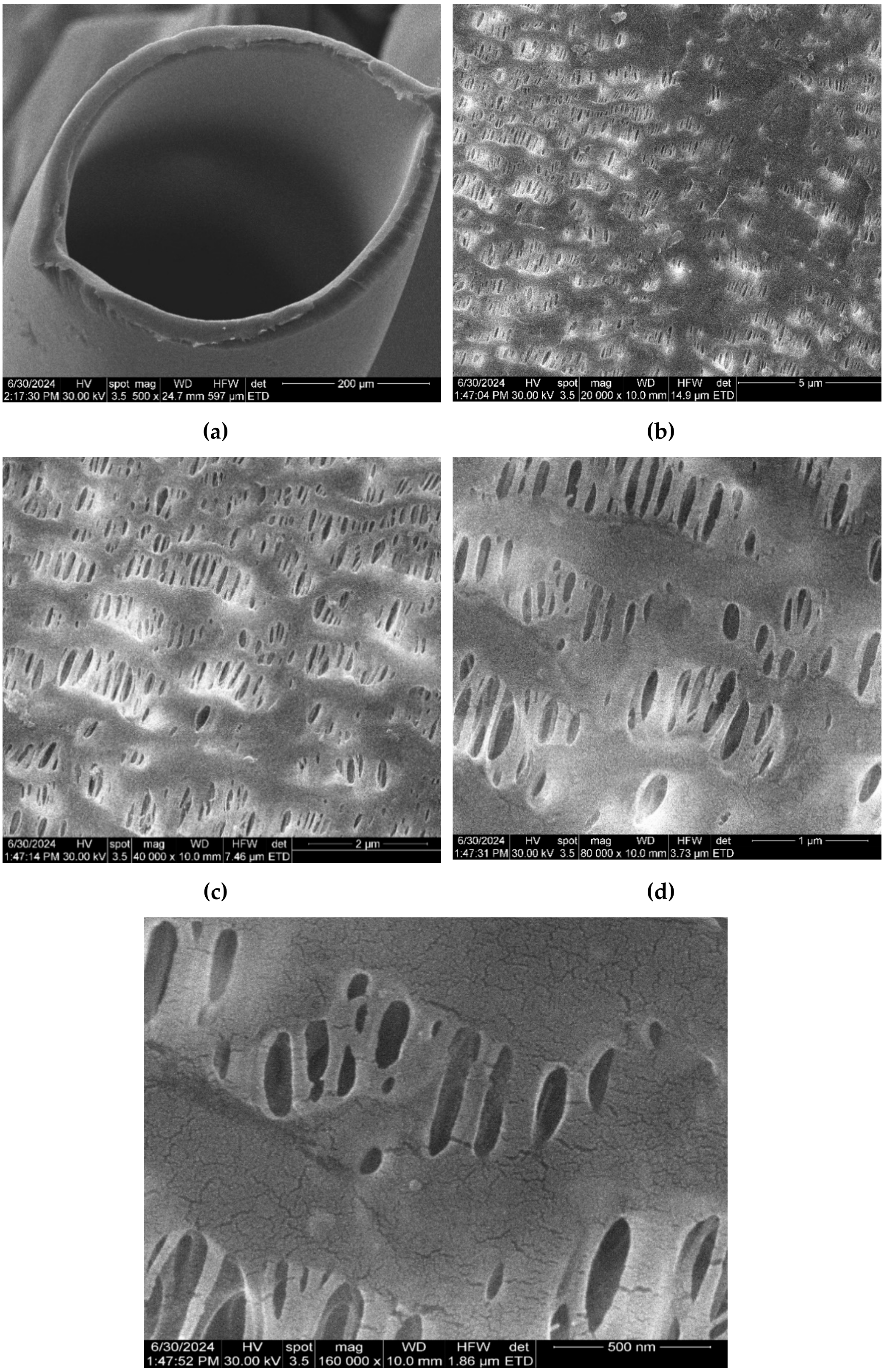

(a) (b) (c) (d) (e)

Figure S3. Scanning electron microscopy for 2–hydroxyethyl–cellulose (HEC)–polypropylene hollow fiber membrane (P4): (**a**) composite membrane section; (**b**) membrane surface at 20,000× magnification; (**c**) membrane surface at 40,000× magnification; (**d**) membrane surface at 80,000× magnification; and (**e**) membrane surface at 160,000× magnification.

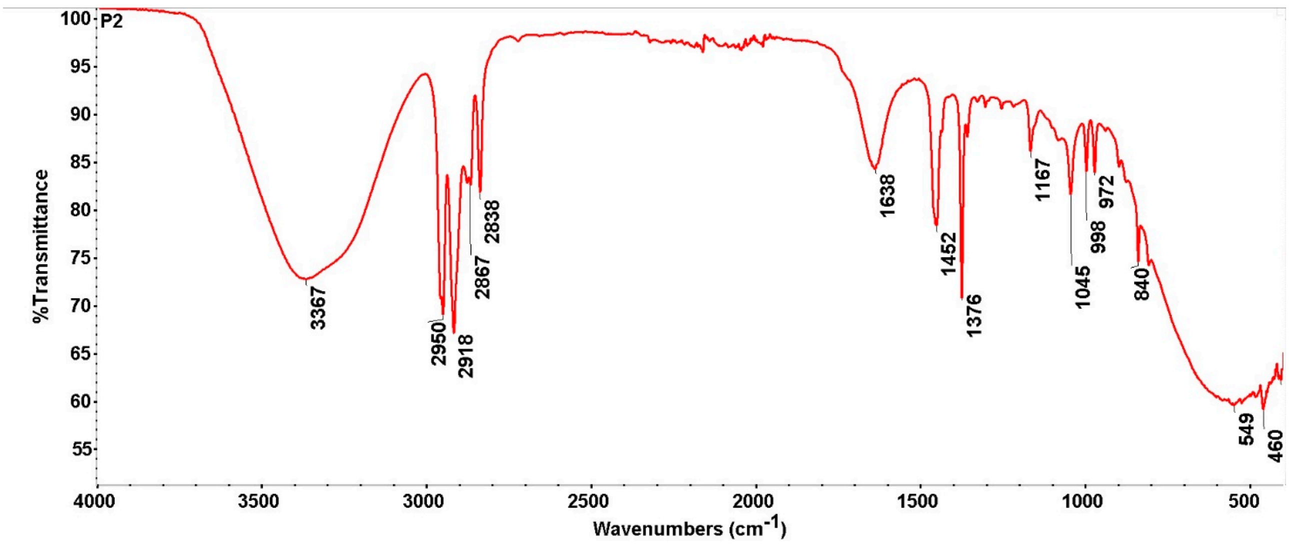

Figure S4. Fourier Transform Infra-Red (FTIR) spectra for P2 composite membrane.

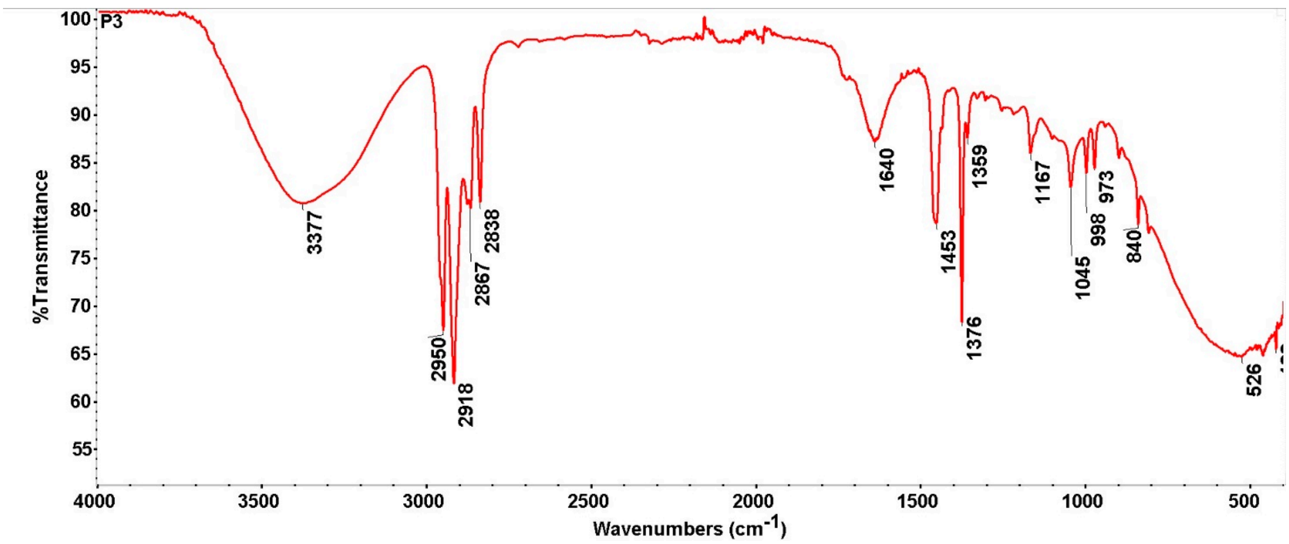

Figure S5. Fourier Transform Infra-Red (FTIR) spectra for P3 composite membrane.

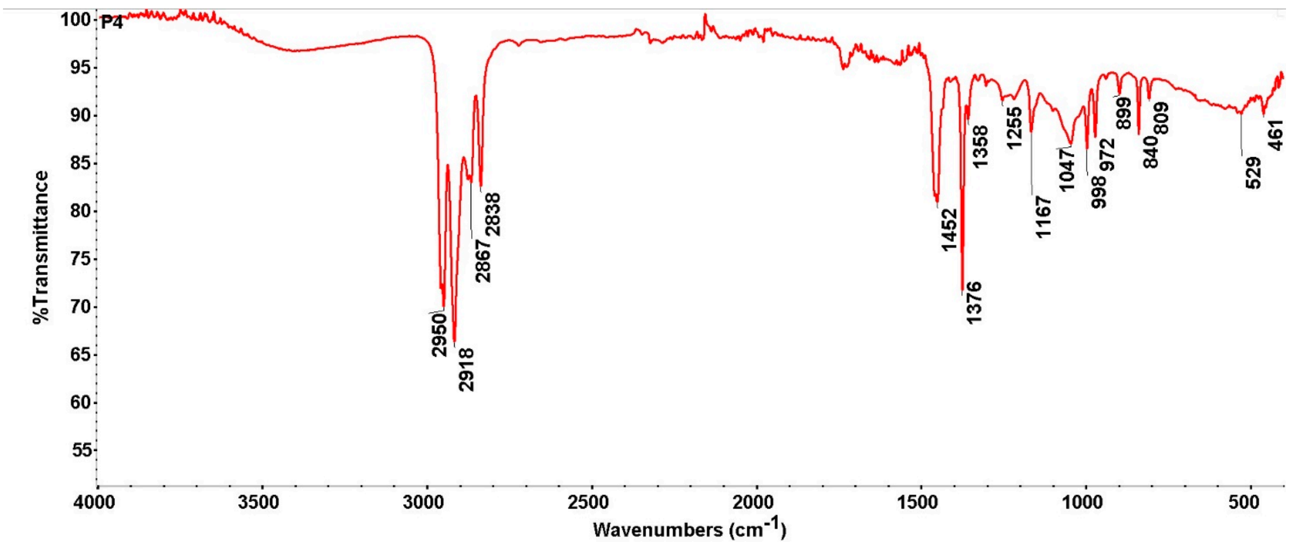

Figure S6. Fourier Transform Infra-Red (FTIR) spectra for P4 composite membrane.

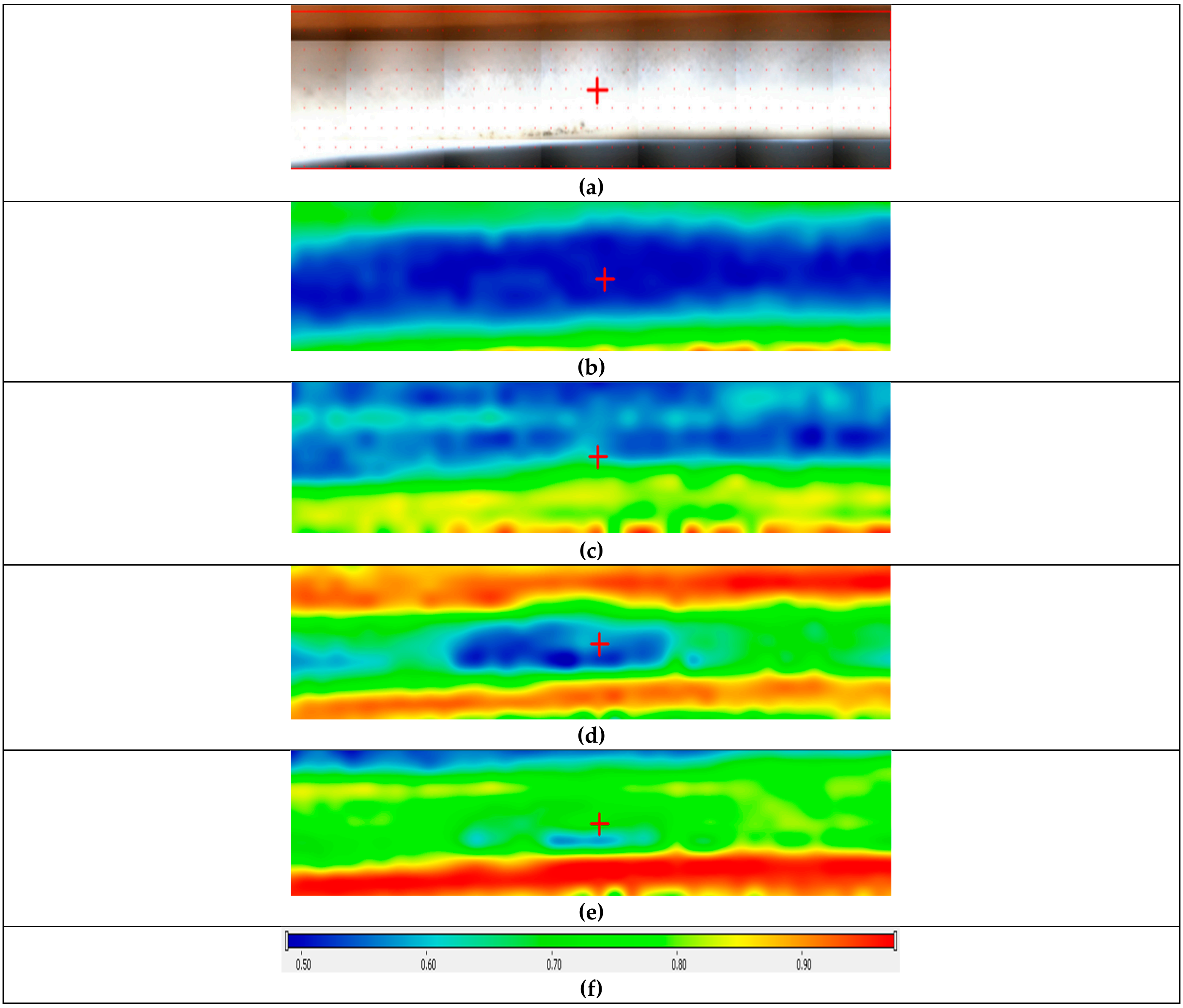

Figure S7. FTIR 2D maps for cellulose acetate–polypropylene hollow fiber membrane: (**a**) the video image; (**b**) 2D image at wavenumber 3386 cm^{-1}; (**c**) 2D image at wavenumber 2950 cm^{-1}; (**d**) 2D image at wavenumber 1639 cm^{-1}; and (**e**) 2D image at wavenumber 1170 cm^{-1}; (**f**) color scale.

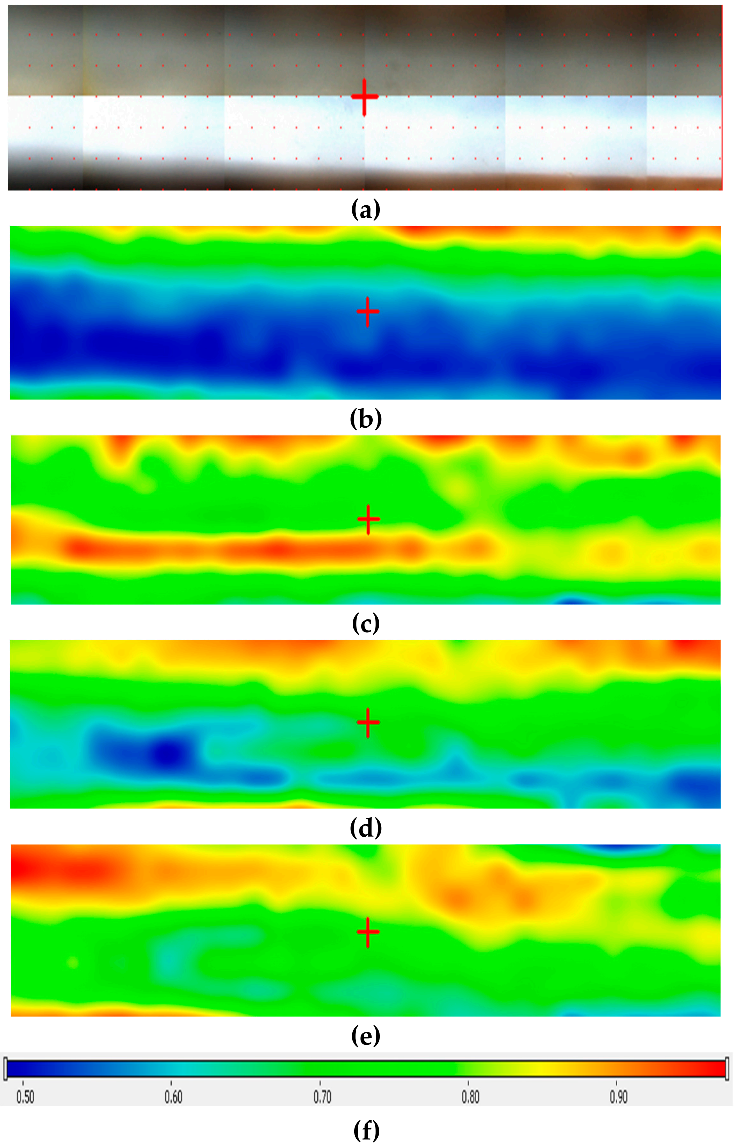

Figure S8. FTIR 2D maps for methyl 2–hydroxyethyl–cellulose –polypropylene hollow fiber membrane: (**a**) the video image; (**b**) 2D image at wavenumber 3386 cm^{-1}; (**c**) 2D image at wavenumber 2950 cm^{-1}; (**d**) 2D image at wavenumber 1639 cm^{-1}; and (**e**) 2D image at wavenumber 1170 cm^{-1}; (**f**) color scale.

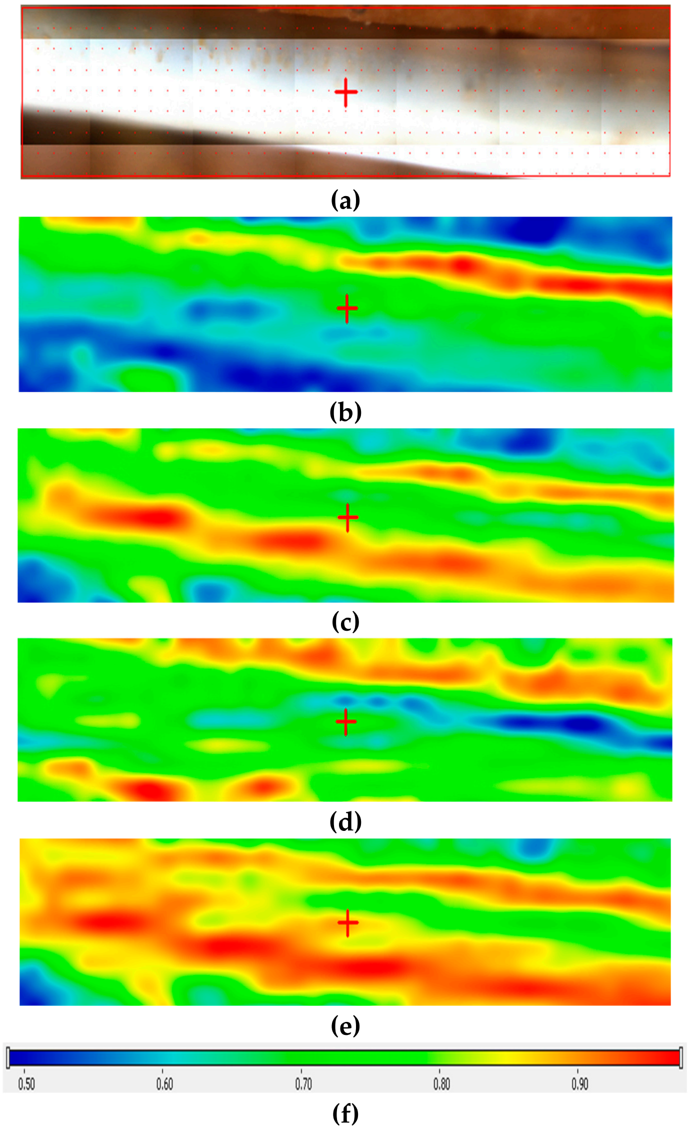

Figure S9. FTIR 2D maps for 2–hydroxyethyl–cellulose –polypropylene hollow fiber membrane: (**a**) the video image; (**b**) 2D image at wavenumber 3386 cm^{-1}; (**c**) 2D image at wavenumber 2950 cm^{-1}; (**d**) 2D image at wavenumber 1639 cm^{-1}; and (**e**) 2D image at wavenumber 1170 cm^{-1}; (**f**) color scale.

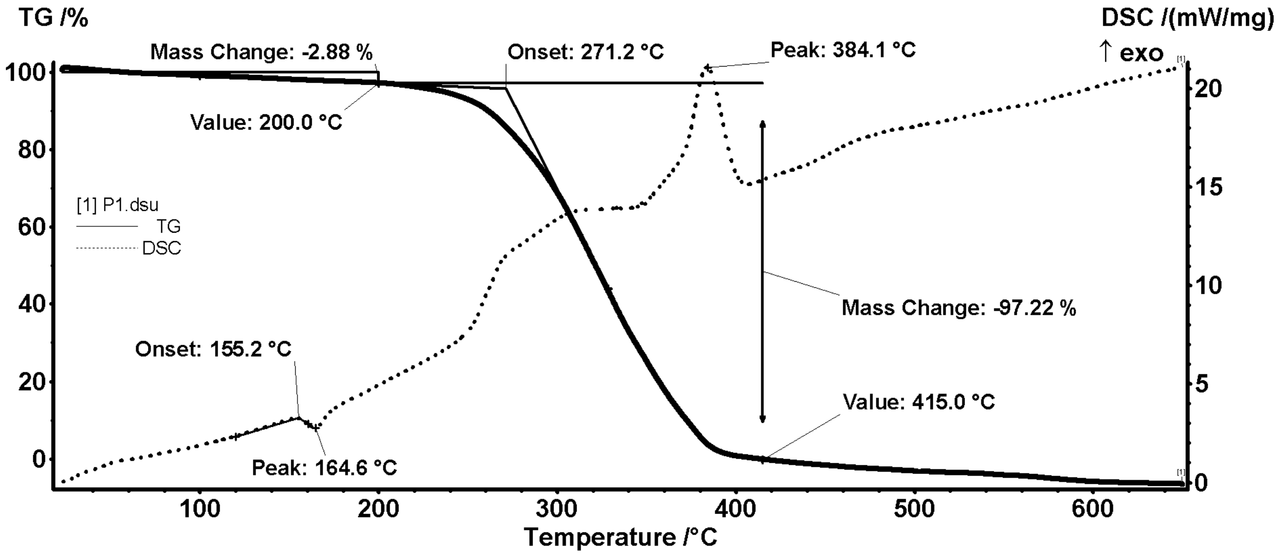

Figure S10. Detail of thermal diagrams for P1.

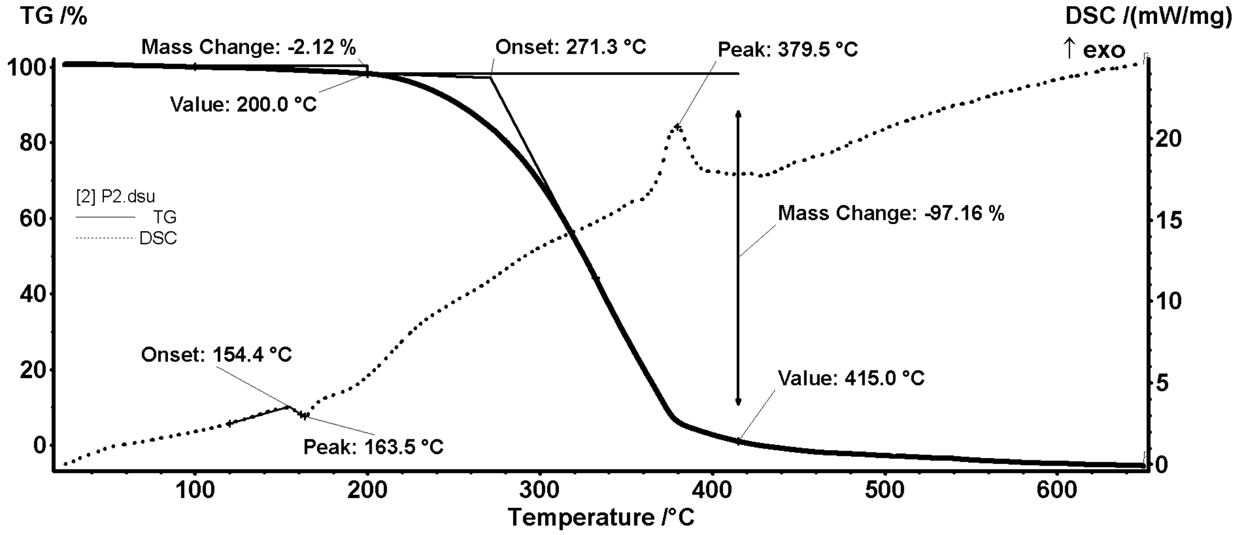

Figure S11. Detail of thermal diagrams for P2.

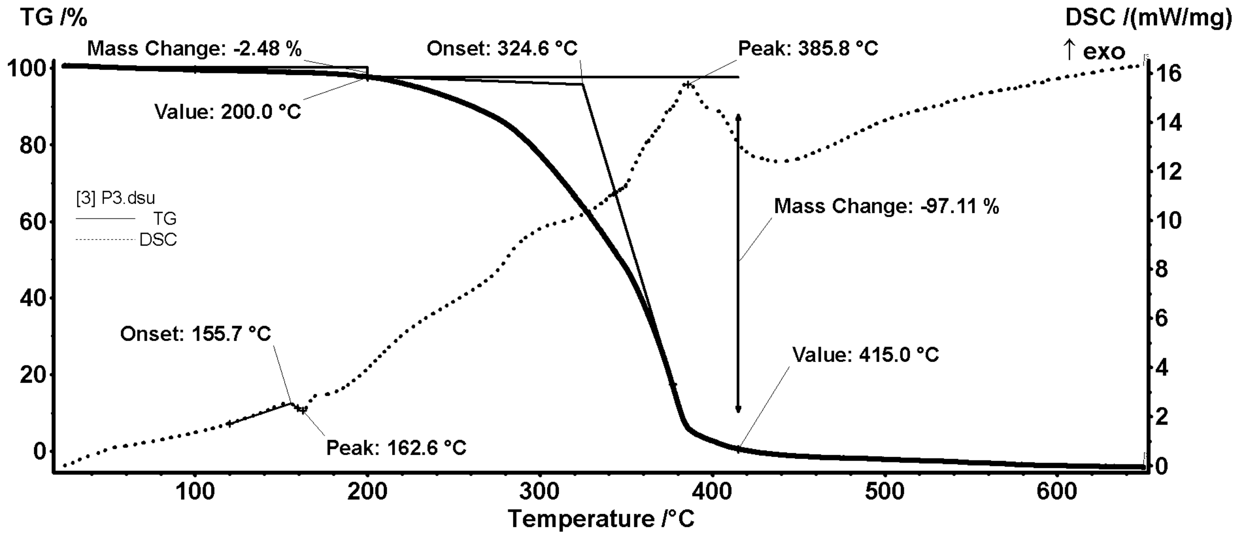

Figure S12. Detail of thermal diagrams for P3.

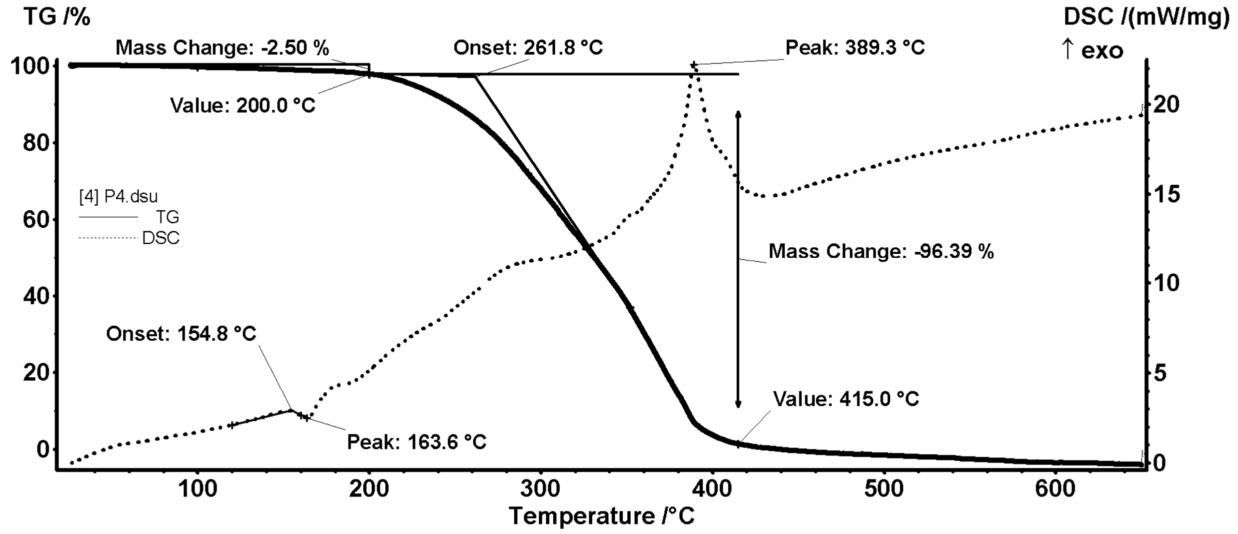

Figure S13. Detail of thermal diagrams for P4.

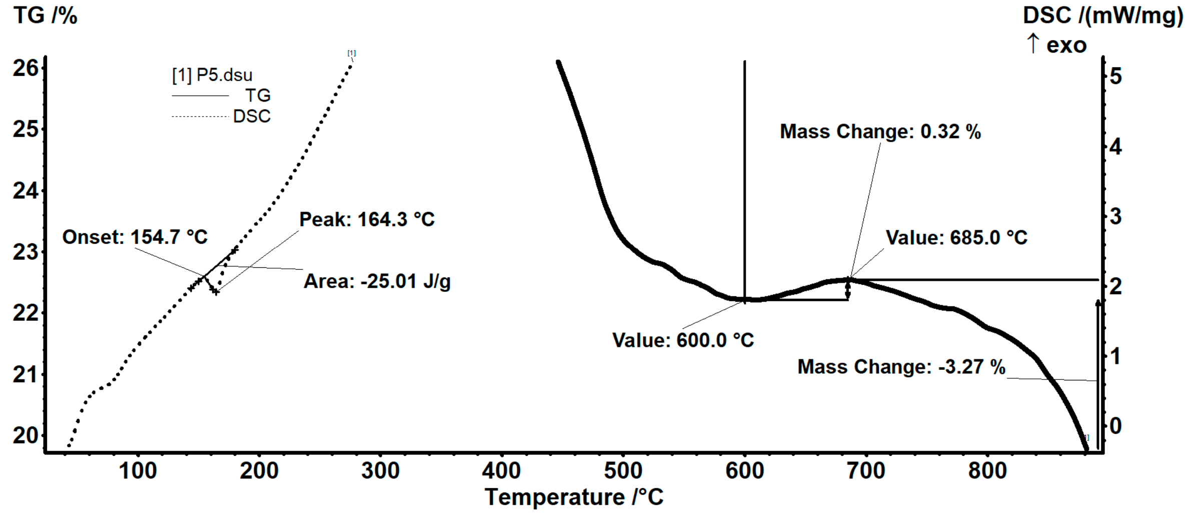

Figure S14. Zoom-in detail for Figure 11 (see the main paper), in case of P5.

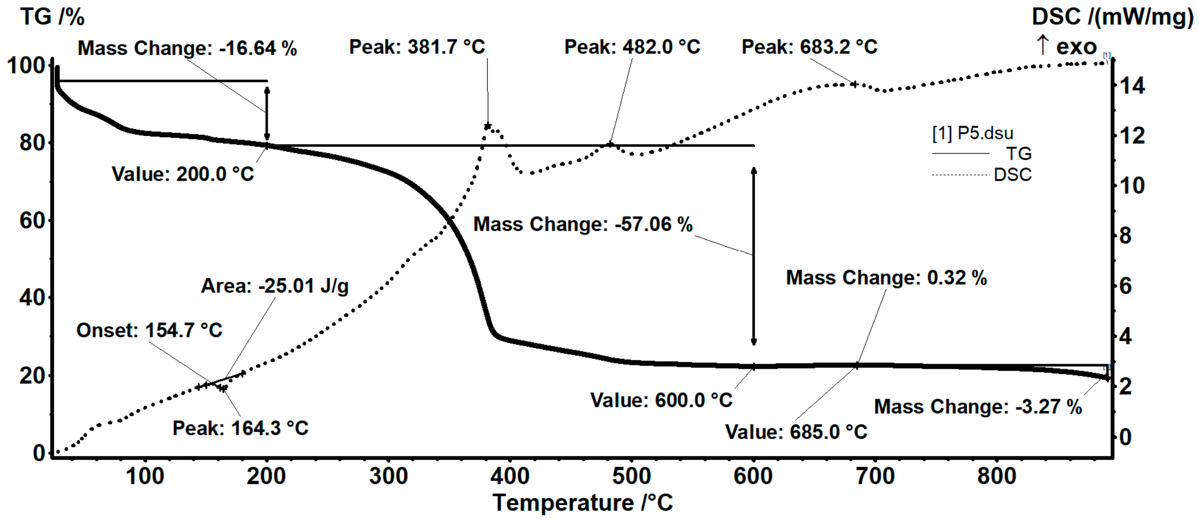

Figure S15. Detail of thermal diagrams for P5.
